# Structural inequality and temporal brain dynamics across diverse samples

**DOI:** 10.1002/ctm2.70032

**Published:** 2024-10-03

**Authors:** Sandra Baez, Hernan Hernandez, Sebastian Moguilner, Jhosmary Cuadros, Hernando Santamaria‐Garcia, Vicente Medel, Joaquín Migeot, Josephine Cruzat, Pedro A. Valdes‐Sosa, Francisco Lopera, Alfredis González‐Hernández, Jasmin Bonilla‐Santos, Rodrigo A. Gonzalez‐Montealegre, Tuba Aktürk, Agustina Legaz, Florencia Altschuler, Sol Fittipaldi, Görsev G. Yener, Javier Escudero, Claudio Babiloni, Susanna Lopez, Robert Whelan, Alberto A Fernández Lucas, David Huepe, Marcio Soto‐Añari, Carlos Coronel‐Oliveros, Eduar Herrera, Daniel Abasolo, Ruaridh A. Clark, Bahar Güntekin, Claudia Duran‐Aniotz, Mario A. Parra, Brian Lawlor, Enzo Tagliazucchi, Pavel Prado, Agustin Ibanez

**Affiliations:** ^1^ Departamento de Psicología Universidad de los Andes Bogota Colombia; ^2^ Global Brain Health Institute (GBHI) University of California San Francisco California USA; ^3^ Global Brain Health Institute (GBHI) Trinity College Dublin Dublin Ireland; ^4^ Latin American Brain Health Institute Universidad Adolfo Ibañez Santiago de Chile Chile; ^5^ Harvard Medical School Harvard University Boston Massachusetts USA; ^6^ Advanced Center for Electrical and Electronic Engineering, Universidad Técnica Federico Santa María Valparaíso Chile; ^7^ Grupo de Bioingeniería, Decanato de Investigación, Universidad Nacional Experimental del Táchira San Cristóbal Venezuela; ^8^ PhD Program in Neuroscience Pontificia Universidad Javeriana Bogota Colombia; ^9^ Center of Memory and Cognition Intellectus, Hospital Universitario San Ignacio Bogotá San Ignacio Colombia; ^10^ Technology of China Chengdu China; ^11^ Cuban Neuroscience Center La Habana Cuba; ^12^ Grupo de Neurociencias de Antioquia, University of Antioquia Medellín Colombia; ^13^ Master Programme of Clinical Neuropsychology, Universidad Surcolombiana Neiva Huila Colombia; ^14^ Department of Psychology Universidad Cooperativa de Colombia Bogota Colombia; ^15^ Neurocognition and Psychophysiology Laboratory Universidad Surcolombiana Neiva Huila Colombia; ^16^ Department of Biophysics School of Medicine Istanbul Medipol University Istanbul Turkey; ^17^ Cognitive Neuroscience Center, Universidad de San Andrés Buenos Aires Argentina; ^18^ National Scientific and Technical Research Council (CONICET) Buenos Aires Argentina; ^19^ Facultad de Psicología, Universidad Nacional de Córdoba Córdoba Argentina; ^20^ School of Psychology, Trinity College Dublin Dublin Ireland; ^21^ Faculty of Medicine, Izmir University of Economics Izmir Turkey; ^22^ Brain Dynamics Multidisciplinary Research Center Dokuz Eylul University Izmir Turkey; ^23^ Izmir Biomedicine and Genome Center Izmir Turkey; ^24^ School of Engineering, Institute for Imaging, Data and Communications, University of Edinburgh Scotland UK; ^25^ Department of Physiology and Pharmacology ‘V. Erspamer’ Sapienza University of Rome Rome Italy; ^26^ Hospital San Raffaele Cassino Cassino Frosinone Italy; ^27^ Department of Legal Medicine Psychiatry and Pathology at the Complutense University of Madrid Madrid Spain; ^28^ Center for Social and Cognitive Neuroscience (CSCN), School of Psychology, Universidad Adolfo Ibáñez Penalolen Chile; ^29^ Universidad Católica San Pablo Arequipa Peru; ^30^ Centro Interdisciplinario de Neurociencia de Valparaíso (CINV), Universidad de Valparaíso Valparaíso Chile; ^31^ Departamento de Estudios Psicológicos Universidad Icesi Cali Colombia; ^32^ Faculty of Engineering and Physical Sciences, Centre for Biomedical Engineering, School of Mechanical Engineering Sciences, University of Surrey Guildford UK; ^33^ Department of Electronic and Electrical Engineering University of Strathclyde Glasgow UK; ^34^ Department of Electronic and Electrical Engineering Centre for Signal and Image Processing University of Strathclyde Glasgow UK; ^35^ Health Sciences and Technology Research Institute (SABITA) Istanbul Medipol University Istanbul Turkey; ^36^ Department of Psychological Sciences and Health University of Strathclyde Glasgow UK; ^37^ University of Buenos Aires Buenos Aires Argentina; ^38^ Escuela de Fonoaudiología, Facultad de Odontología y Ciencias de la Rehabilitación, Universidad San Sebastián Santiago Chile; ^39^ Trinity College Dublin, The University of Dublin Dublin Ireland

**Keywords:** brain dynamics, cognition, demographics, EEG, individual differences, structural income inequality

## Abstract

**Background:**

Structural income inequality – the uneven income distribution across regions or countries – could affect brain structure and function, beyond individual differences. However, the impact of structural income inequality on the brain dynamics and the roles of demographics and cognition in these associations remains unexplored.

**Methods:**

Here, we assessed the impact of structural income inequality, as measured by the Gini coefficient on multiple EEG metrics, while considering the subject‐level effects of demographic (age, sex, education) and cognitive factors. Resting‐state EEG signals were collected from a diverse sample (countries = 10; healthy individuals = 1394 from Argentina, Brazil, Colombia, Chile, Cuba, Greece, Ireland, Italy, Turkey and United Kingdom). Complexity (fractal dimension, permutation entropy, Wiener entropy, spectral structure variability), power spectral and aperiodic components (1/*f* slope, knee, offset), as well as graph‐theoretic measures were analysed.

**Findings:**

Despite variability in samples, data collection methods, and EEG acquisition parameters, structural inequality systematically predicted electrophysiological brain dynamics, proving to be a more crucial determinant of brain dynamics than individual‐level factors. Complexity and aperiodic activity metrics captured better the effects of structural inequality on brain function. Following inequality, age and cognition emerged as the most influential predictors. The overall results provided convergent multimodal metrics of biologic embedding of structural income inequality characterised by less complex signals, increased random asynchronous neural activity, and reduced alpha and beta power, particularly over temporoposterior regions.

**Conclusion:**

These findings might challenge conventional neuroscience approaches that tend to overemphasise the influence of individual‐level factors, while neglecting structural factors. Results pave the way for neuroscience‐informed public policies aimed at tackling structural inequalities in diverse populations.

## Graphical abstract

We analysed EEG data from 1394 participants across 10 countries, using the Gini coefficient and sociodemographic variables to predict EEG metrics.

Four categories of EEG metrics were computed: complexity, aperiodic spectral components, power spectrum, and connectivity.

ROC curves, feature importance rankings, and topographical brain region information were reported.

Structural income inequality consistently predicts EEG metrics, surpassing individual demographic factors.

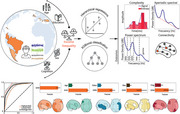

## 1 INTRODUCTION

Structural income inequality – the uneven income distribution among individuals or household within regions or countries^1^, ^2^ – plays a major role in health outcomes,^3^, ^4^ beyond individual‐level differences. Similarly, structural socioeconomic inequalities may affect brain health.^5^, ^6^, ^7^, ^8^, ^9^, ^10^ However, most research has traditionally focused on individual level factors,^11^, ^12^, ^13^, ^14^ linking magnetic resonance imaging (MRI) and functional MRI (fMRI) measures to lower socioeconomic status,^15^, ^16^, ^17^ education,^15^, ^16^, ^18^ and personal income.^15^, ^16^, ^18^ A few electroencephalography (EEG) developmental studies have identified associations with family income^19^, ^20^, ^21^, ^22^ and maternal education.^19^ Individual income correlates with EEG complexity in adulthood,^23^ while in older individuals, perceived socioeconomic status is related to changes in power spectrum values.^24^ Recent calls^25^, ^26^ suggest that structural income inequality, as measured by the Gini coefficient,^10^, ^27^ may impact brain dynamics. Despite this growing interest in the association between the Gini coefficient and brain health, evidence is still emerging,^1^ highlighting changes in brain structure in adolescents,^28^ mental health symptoms,^29^, ^30^, ^31^, ^32^ subjective cognitive decline,^33^ allostatic overload,^34^ and Alzheimer's disease prevalence.^35^ Nevertheless, the effects of structural income inequality on different properties of the brain's temporal dynamics remain understudied.

The idea of biologic embedding of structural inequalities^1^, ^36^ has gained support with evidence showing a link with brain structure^37^, ^38^, ^39^ and function.^40^, ^41^ Country‐level gender inequality correlates with differences in cortical thickness between women and men.^37^ State‐level inequality is linked to reduced hippocampal volume in youths.^38^ Neighbourhood‐level income inequality is associated with functional brain network development during adolescence,^40^ and lower brain volumes in aging.^39^ However, the impact of country‐level income inequality on brain dynamics, especially in diverse populations, remains unclear. Previous research has primarily focused on high‐income countries,^38^, ^39^, ^40^, ^41^ neglecting the large structural disparities in the global south,^5^ thereby limiting the generalisability of findings.^25^ To understand how societal disparities impact brain outcomes, it is crucial to include measures of structural inequality at the country level, especially in nations with greater inequalities.^36^ Multi‐country studies encompassing diverse levels of structural income inequality are essential for this framework.

Electroencephalography is a suitable method for large‐scale studies in global and unequal contexts given its non‐invasiveness, scalability, temporal resolution, affordability, and measurement of direct brain activity.^42^ Despite its potential, no EEG study has incorporated structural inequality as a measure. Furthermore, current evidence on structural inequality and brain associations lacks an understanding of how individual factors, including demographic (age, sex, education) and cognitive factors influence these links. Altough demographic variables such as sex or education, do not constitute measures of inequality, are linked to disparities.^36^, ^43^, ^44^ Assessing them alongside country‐level structural inequality can provide a more comprehensive understanding of their biological embedding.

Considering these gaps, we investigated the effect of country‐level structural income inequality, as measured by the Gini coefficient, on multiple EEG metrics while considering the effects of demographic (age, sex, and education) and cognitive factors. Resting‐state EEG signals were collected from a large sample of healthy individuals (*N* = 1394) from the global south and north (Argentina, Brazil, Colombia, Chile, Cuba, Greece, Ireland, Italy, Turkey, United Kingdom). We included EEG complexity, spectral activity (power spectrum and aperiodic spectral components), and graph‐theoretic measures. We selected these metrics given their relevance for individual‐level factors (i.e., age,^45^, ^46^, ^47^ sex,^48^, ^49^, ^50^ education,^20^ and cognition^51^, ^52^, ^53^, ^54^), their potential implications for understanding the effects of inequality on brain function, and their frequent use in previous studies. Hierarchical regressions were performed to test whether inequality could significantly predict specific EEG metrics after adjusting for demographic and cognitive factors. Then, we evaluated the power of structural income inequality (the Gini coefficient), both individually and in combination with demographic and cognitive factors, to classify these metrics. This approach allowed us to evaluate the unique contribution of structural income inequality to EEG outcomes while controlling for individual differences. We developed a set of hypotheses based on previous MRI^37^, ^38^, ^39^ and fMRI^40^, ^41^ studies and changes in the EEG metrics^19^, ^20^, ^22^, ^23^, ^24^ that were associated with individual‐level income inequality. We hypothesised that larger structural inequality would significantly affect temporal brain dynamics, such as reduced power spectrum values, aperiodic activity, and EEG complexity. Similarly, higher inequality would be associated with decreased integration and increased network segregation.^55^ The association between structural inequality and EEG metrics would remain significant even after accounting for individual‐level variations (demographics and cognition). These effects would not be explained by differences in data quality, number of EEG channels, or sample size variations. Overall, these hypotheses aimed to test the biological embedding of macrosocial structural income inequality beyond individual (demographic and cognitive) variation across multiple brain temporal dynamics.

## 2 MATERIALS AND METHODS

### 2.1 Participants

**FIGURE 1:**
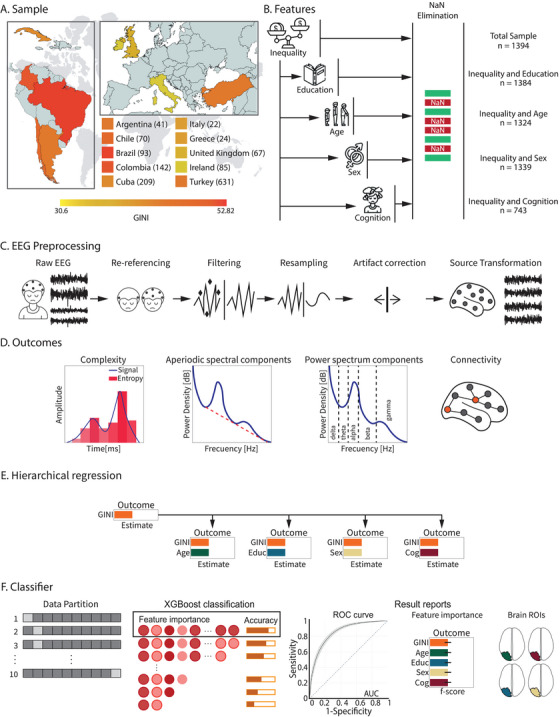
An integrative approach for assessing EEG correlates of income inequalities. (A) Geographical distribution of the sample. The participating countries are highlighted. Numbers in parentheses indicate the sample sizes for each country. The colour bar emphasises the Gini coefficients. (B) Sociodemographic variables. The set of variables explored includes inequality, demographics and cognition. Education data were available for 1384 participants; sex information was provided by 1339 individuals, and 743 participants were assessed for cognition. (C) EEG preprocessing: The main steps of the processing workflow. (D) EEG outcomes. Four categories of metrics were computed in the EEG source space: complexity components, aperiodic spectral components, power spectrum components, and connectivity. (E) Hierarchical regressions. Associations were assessed between sociodemographic variables and EEG outcomes. Linear regression models were implemented using Gini as a predictor, as well as in combination with demographics and cognition, respectively. (F) Classifiers. The XGBoost classifier was utilised with an 80% training sample and a 20% testing set (*k* = 10 repetitions) to explore the predictive value of inequality and its interaction with demographics and cognition for EEG outcomes. Classification models tuned through cross‐validation employing five sets of predictors: Gini, Gini and years of education, Gini and age, Gini and sex, and Gini and cognition. We reported ROC curves, rankings of feature importance, and topographical information about representative brain regions.

This multicentre study involved 1394 healthy participants (age: *M* = 46.44 SD = 20.93, range = 17−91; years of education: *M* = 13.77, SD = 4.38, range = 0−28; sex: male = 635, female = 714) from countries with different levels of income inequality, according to the World Bank classification. These countries belong to the global south (Argentina, Brazil, Chile, Colombia, and Cuba) and the global north (Greece, Ireland, Italy, United Kingdom and Turkey), as defined by the United Nations Conference on Trade and Development. Table 1 and Figure 1A show the demographic characteristics and the geographic distribution of the datasets. Education data were available for 1384 participants; 1339 individuals with demographics, and cognition was assessed in 743 participants. Participants reported no history of psychiatric or neurological disorders, alcohol/drug abuse, and significant visual and/or auditory impairments. No participants reported cognitive complaints or functional impairments. All procedures were performed in accordance with the ethical standards of the Helsinki Declaration and its later amendments. The Institutional Ethics Committee at each participating centre approved the study protocol, and all participants provided written informed consent.

**TABLE 1:** Demographic and cognitive information of the participants.

		Sex		Age		Education		Cognition		
Socioeconomic region	Country	Sex	*n*	Mean	SD	Mean	SD	*n*	Mean	SD
Global south	Argentina	Female	29	66.76	8.53	17.03	2.03	28	29.04	.69
		Male	12	66.33	7.92	15.42	2.94	12	28.17	1.85
	Chile	Female	59	57.03	17.10	15.49	3.73	49	28.80	1.47
		Male	21	62.62	18.46	14.33	4.35	14	28.30	2.27
	Brazil	Female	58	59.33	16.88	11.88	3.68	58	28.03	1.61
		Male	35	62.69	15.91	13.51	2.34	35	28.29	1.51
	Colombia	Female	96	49.61	13.85	12.43	4.64	96	27.82	3.03
		Male	37	45.49	15.49	12.68	4.59	37	28.00	3.13
	Cuba	Female	53	37.51	11.34	13.25	2.79	31	29.68	.70
		Male	149	29.68	7366	12.99	2.81	94	29.28	1.42
Global north	Italy	Female	12	60.55	7.35	15.25	4.85	–	–	–
		Male	9	62.90	9.24	15.44	5.46	–	–	–
	Greece	Female	10	68.90	5.34	–	–	10	30	0
		Male	14	68.64	5.43	–	–	14	30	0
	United Kingdom	Female	34	56.82	17.40	15.00	3.67	–	–	–
		Male	19	53.89	21.13	15.11	10.27	–	–	–
	Ireland	Female	41	65.76	4.54	15.50	3.78	40	29.25	.90
		Male	44	69.41	5.85	15.01	4.17	44	28.75	1.10
	Turkey	Female	319	43.48	22.02	13.37	4.98	118	28.85	1.10
		Male	271	39.53	22.03	14.19	4.12	69	29.10	1.36

The power analysis with G*Power 3.1.9.7 software,^56^ indicated that the smallest subsample of our study, consisting of 743 participants with cognitive measurements, exceeded the necessary minimum to sense small effect sizes when conducting multiple linear regressions with a statistical power of .94.

### 2.2 Aggregate‐ and individual‐level predictors

#### 2.2.1 Country‐level income inequality

We used the Gini coefficient,^57^, ^58^, ^59^ a statistical metric depicting the deviation of income distributions within an economy from a perfectly equal distribution.^60^ The Gini coefficient has been crucial in operationalising structural income inequality in numerous cross‐national studies.^32^, ^35^, ^61^, ^62^, ^63^ This coefficient is expressed as the percentage of the area between the Lorenz curve and a 45‐degree line symbolising perfect equality.^64^ The Gini coefficient is calculated as follows:

(1) $$\mathrm{Gini}\;=\;100\times (1-\;\sum_{i\;=\;1}^{N}\left(x_{i}-\;x_{i-1}\right)\left(y_{i}+y_{i-1}\right))\;$$

Here $x_{i}$ and $y_{i}$ are coordinates on the x‐axis and y‐axis, respectively, with the *x*‐axis divided into *N* equal segments. The Gini coefficient provides a single value ranging from 0 to 1, where 0 represents perfect equality and 1 represents maximum inequality.^64^ This coefficient is inherently standardised, allowing for the comparison of income inequality across different countries. Here, we used the Gini coefficient, expressed as a percentage. For our study, the GINI coefficient for each country was computed as the average value for the years during which data were collected,^10^, ^37^ a choice made due to the GINI index's relative stability over successive years. The country‐specific GINI values were obtained from the World Bank's Poverty and Inequality Platform, based on primary household survey data from national statistical offices and World Bank country divisions (www.pip.worldbank.org). For Cuba, the GINI coefficient was as reported by the Economic Commission for Latin America and the Caribbean (CEPAL, 1999).

#### 2.2.2 Demographics

Participants' demographic information includes age at which assessments were conducted (measured in years), sex (classified as male or female), and years of formal education.

#### 2.2.3 Cognition

The overall cognitive state was evaluated using the raw total score derived from the Mini‐Mental State Examination (MMSE).^65^ This tool is a well‐recognised screening, providing an initial assessment of general cognitive functioning. The MMSE ranges from 0 to 30, with scores ≥ 24 indicating normal cognitive functioning.^66^, ^67^, ^68^, ^69^, ^70^

### 2.3 EEG acquisition, processing and harmonization

#### 2.3.1 EEG acquisition

Eyes‐closed resting‐state EEG (rsEEG) was acquired at least for 5 min. The sensor type, signal calibration, and electrode placement configuration varied across centres, as outlined in Table S1. Occasionally, eyes‐open EEG was acquired. As the recording length varied across centres, the EEG analysis was restricted to the initial 5 min of recording.

#### 2.3.2 Preprocessing

Offline preprocessing of EEG data involved a customised and automated pipeline, which included harmonisation protocols to mitigate batch effects and methodological variations commonly found in multi‐centre EEG studies.^71^, ^72^ The workflow encompassed data preprocessing, rescaling, spatial normalisation of EEG records, and source estimation. The EEG was filtered between .5 and 40 Hz using an 8th‐order zero‐phase shift Butterworth filter. In the pipeline employed in this study, sampling frequency and reference selection were less influential factors. EEG recordings were sampled following the Nyquist Theorem, which determined the resolution used in the spectral representation with a sampling frequency of 512 Hz and a 2‐s analysis window. All recordings were referenced using the reference electrode standardisation technique (REST),^73^ which has been shown to result in lower localiszation errors for EEG point sources.^74^

Blinks, eye movements artefacts and myogenic activity were removed using two algorithms^75^: ICLabel, a tool classifying EEG independent components into signals and various noise categories,^76^ and EyeCatch, a tool specifically designed for identifying eye‐related ICA scalp maps.^77^ After artefact correction, a visual inspection of the EEG was conducted. Malfunctioning channels were replaced using weighted spherical interpolations.^78^

#### 2.3.3 EEG normalisations

One critical normalisation step consisted of addressing cross‐site amplitude differences. To minimise variability, Z‐score transformations of EEG time series were implemented.^79^, ^80^ This process involved calculating the mean voltage of each EEG channel and Z‐transforming the voltage samples using the channel's mean and standard deviation. This normalisation effectively minimises variability across electrodes and was carried out separately for each recruitment centre, thereby mitigating differences between sites. Additionally, the EEG data underwent spatial normalisation to align diverse electrode configurations within a unified topographical framework. This was done by mapping the EEG onto a 6067‐point mesh head model^78^, ^81^ and projecting the signal onto a Biosemi 128‐electrode layout.^82^ The spatial normalisation transformed lower‐density electrode data into a high‐density configuration, minimising the impact of electrode layout on EEG source estimation. Reducing variability in channel numbers is crucial for harmonisation.^42^, ^71^ This approach has been successfully applied across different density electrode configurations.^81^ However, newly created channels contain redundant information from existing channels, offering no additional spatial resolution. Thus, the analysis was limited to the 10 ROIs described in Section 2.5.

#### 2.3.4 EEG source estimation

The source space analysis followed a well‐established framework^83^, ^84^ involving a linear conversion of sensor‐level data augmented with anatomical information, to reduce the noise, provide a comes source spaces across datasets, and enhance the interpretability. The EEG source generators were estimated using the standardised Low‐resolution Electromagnetic Tomography Analysis (sLORETA).^83^ This method computes the standardied current density in 2394 virtual sensors and allows the localisation of point sources in both the cerebral cortex and hippocampus^83^, ^85^ of a standard brain (MNI 305, Brain Imaging Centre, Montreal Neurologic Institute).^86^ Standardised current densities encompassed the 5‐min duration of rsEEG sampled at 512 Hz. It overcomes challenges in estimating deep EEG sources and ensuring accuracy despite correlation among nearby generators.^87^ Current densities were estimated for each of the 153 600 voltage distributions comprising the 5 min of rsEEG. The brain was segmented into 82 brain regions using the Automated Anatomical Labeling (AAL) atlas.^88^ Standardised current densities time series estimated in voxels belonging to the same AAL regions were averaged, so a single time series was assigned to each anatomical region.^82^, ^89^, ^90^

### 2.4 EEG outcomes

We computed four categories of outcomes from the source space resting‐state EEG: (i) complexity metrics (Wiener entropy, permutation entropy, spectral structural variability and fractal dimension), (ii) aperiodic spectral properties (1/*f* slope, knee, offset), (iii) power spectral components, and (iv) graph‐theoretical metrics (global efficiency, transitivity, density, and small‐worldness) (Figure 1D). Power spectrum metrics establish a baseline for typical spectral activity, serving as a valuable benchmark for identifying pathological conditions.^91^ The aperiodic spectral components of EEG represent nonrhythmic brain activity, which facilitates physiological interpretations related to aging and cognition.^92^ Complexity metrics enable the evaluation of dynamical brain complexity, which has been explored as potential biomarkers for brain health disorders.^93^, ^94^ Lastly, graph‐theoretic metrics shed light on the structure of functional connectivity patterns and network organisation.^51^ These metrics are associated to individual‐level inequality, demographic and cognitive factors. Individual income is associated with EEG complexity^23^ and changes in EEG power spectrum and aperiodic activity.^19^, ^20^, ^22^, ^24^ Age, education, sex and cognition are also linked with complexity metrics,^95^, ^96^, ^97^, ^98^ power spectrum values,^45^, ^46^, ^49^, ^53^, ^54^, ^99^ aperiodic spectral activity,^45^, ^49^, ^99^, ^100^ and graph‐theoretic EEG measures.^51^, ^52^, ^53^, ^54^, ^101^, ^102^

#### 2.4.1 Complexity metrics

This study incorporated four complexity metrics: Fractal Dimension (FD), Permutation Entropy (PE), Wiener Entropy (WE), and Spectral Structure Variability (SSV). The FD (Table S2, Panel 2.1) is a statistical indicator of intricacy within patterns, offering a way to quantify the randomness or fragmentation in EEG signals across different scales.^103^, ^104^, ^105^ The PE (Table S2, Panel 2.2) is a nonparametric, ordinal‐based measure reflecting the temporal ordering structure in time series^103^, ^104^, ^106^ that indexes the predictability of the EEG signal. The WE, or spectral flatness (Table S2, Panel 2.3), measures the uniformity of a signal's energy distribution across the frequency domain,^103^, ^104^, ^107^ and distinguishes signals resembling sinusoidal functions from those akin to noise, providing insight into signal regularity. The SSV (Table S2, Panel 2.4) examines changes in spectral characteristics over time or space, highlighting the dynamics within the EEG signals.^107^

The interpretation of the complexity metrics depend on the range of values under study. For low values, these metrics indicate a highly regular signal with low information content, while the largest values represent irregular and noisy signals, such as white noise with a flat power spectrum. Values in between these two extremes are characteristic of complex signals at the critical transition between order and disorder, which are indicative of complex information processing in the brain.^108^ To prevent segmenting the analyses with complexity metrics, we standardised low complexity values (values below the mean minus 1.2 times the standard deviation) and high complexity values (values above the mean plus 1.2 times the standard deviation). Subsequently, we reversed the standardised high complexity values to create a uniform scale of complexity. This scale indicates that low complexity values are linked to atypical brain activities (low information signals and irregular and noisy signals),^104^, ^109^, ^110^ while high values represent normal brain function.^111^

#### 2.4.2 EEG aperiodic spectral components

EEG aperiodic spectral components were computed through the estimation of the 1/*f* slope, knee, and offset within the EEG power spectral density (PSD)^112^, ^113^, ^114^ (details in Table S3). Application of the fitting oscillations and one‐over‐F (FOOOF) algorithm was executed on the PSD, spanning the frequency range from .5 to 40 Hz.^112^, ^113^, ^114^ The FOOOF algorithm is explicitly designed for the identification and modelling of characteristics within the PSD,^92^ employed a Lorentzian function to describe the aperiodic component, as follows:

(2) $$A\;=\;b-\log\left(k+F^{x}\right),\;$$

where *A* is the aperiodic component, *b* is the offset, *x* is the exponent, and *x = –a*.

#### 2.4.3 EEG power spectrum components

The analysis of the power spectrum components of the EEG was conducted in both the canonical EEG frequency bands and the subject‐specific EEG frequency bands^115^ (see Table 4). The canonical frequency bands were defined as follows: delta (𝛿): 1.5‐6 Hz, theta (𝛳): 6.5–8.0 Hz, alpha1 (𝛼1): 8.5–10 Hz, alpha2 (𝛼2): 10.5–12.0 Hz, beta1 (𝛽1): 12.5–18.0 Hz, beta2 (𝛽2): 18.5–21.0 Hz, beta3 (𝛽3): 21.5–30.0 Hz, and gamma (𝛾): 30.0–40.0 Hz.^115^ These band ranges have been applied in EEG source‐space spectral analysis for assessing dementia^82^, ^116^ and other conditions.^117^, ^118^, ^119^, ^120^, ^121^

The subject‐specific frequency bands were determined by identifying two landmarks in the EEG spectrum, the individual alpha peak frequency (IAF) and the theta/alpha‐frequency transition (TF). While the IAF represents the EEG frequency with maximum power in the alpha‐frequency band, the TF indicates the EEG frequency with minimum power in the second half of the theta frequency range.^115^, ^122^, ^123^ The subject‐specific frequency bands were defined as: 𝛿 (TF‐4 to TF‐2), 𝛳 (TF‐2 to TF), 𝛼low (TF to IAF), and 𝛼high (IAF to IAF+2).^122^ As expected, the 𝛽 and 𝛾 frequency bands corresponded to the canonical division.

We calculated both the power spectral density (PSD) and the normalised PSD (nPSD) using Welch's method with 1‐s Hanning windows and a 50% overlap. Subsequently, the mean of the nPSD within each frequency band (equivalently per cent power)^124^ and the percentage of the nPSD of a given frequency band relative to the total nPSD (relative power density)^115^, ^125^ were computed.

#### 2.4.4 Graph‐theoretic measures

Whole‐brain functional network analyses were conducted using graph‐theoretic measures.^82^, ^90^ We employed the Gaussian copulas approximation,^122^ a robust computational framework that integrates statistical theory with a definitive solution for the entropy of multivariate Gaussian distributions. The connectivity metrics were mutual information (MI), conditional mutual information (CMI), and organisational information (O_info)^82^, ^90^, ^122^, ^126^ (see Supplementary Information S1). The functional network matrices were used to conduct undirected weighted graph‐based network analyses of the EEG source space. Metrics of segregation, integration and global network organisation were calculated (see Table S4).

### 2.5 Reduction of the EEG analytical space

Two complementary strategies were implemented in the analysis of spectral EEG outcomes.^127^ Firstly, we merged anatomically and functionally related AAL regions to create cohesive regions of interest (ROIs, Table S5). When all anatomical regions from the AAL atlas were merged, we designated the resulting ROI as ‘all ROIs’. Then, the EEG analytical space was further reduced using statistical criteria.

#### 2.5.1 Region of interest selection

We merged regions from the AAL atlas to create 10 cohesive ROIs (Table S5). In addition, for specific metrics, like entropy, certain regions cover the entire brain, which will be labelled as ‘all ROIs’ henceforth. This approach involved two critical criteria: (i) bundling together brain regions associated with a specific cortical gyrus (e.g., superior, middle, and inferior orbital gyri) into a ROI and (ii) grouping neighbouring regions with established functional coupling, such as the Rolandic operculum and insula. The proposed method simplifies EEG analysis by focusing on ROIs encompassing both structurally and functionally related regions, rather than examining each region in isolation. This simplification enhances data interpretation and aids identifying patterns or significant features in brain activity.

#### 2.5.2 Statistical reduction of complexity and spectral features

To refine the analytical space of spectral and complexity metrics, statistical criteria were applied. ROIs demonstrating statistically significant relative power density and equivalent per cent power^125^, ^128^, ^129^ were identified through mean‐vs‐zero nonparametric permutation tests [(α = .05; 5000 randomisations^130^]. This step, applied to both canonical and individual EEG band classifications,^115^ was carried out for each frequency band. The results were then adjusted for multiple comparisons using the Benjamini and Hochberg False‐Discovery Rate (FDR) method. ROIs representing the Individual Alpha Frequency (IAF)^129^, ^131^ were those exhibiting statistically significant α activities. Similarly, ROIs indicative of the θ‐α transition (TF)^129^, ^131^ were those with statistically significant θ activity. Likewise, the analytical space of aperiodic^112^, ^113^, ^114^ and complexity^103^, ^104^ metrics was further reduced by implementing mean‐vs‐zero nonparametric permutation tests, followed by correction using the Benjamini and Hochberg FDR method.

### 2.6 EEG features classification and prediction from the Gini coefficient, demographics and cognition

#### 2.6.1 Hierarchical regressions

We employed hierarchical regressions to assess whether inequality significantly predicts EEG metrics and to examine the consistency of its effects in conjunction with each demographic or cognitive factor. We identified the metrics that were significantly associated with structural inequality. Subsequently, for cases where inequality had a significant impact (∼75% of the metrics), we applied XGBoost classifiers to determine the predicted power of inequality on EEG metrics, and to provide feature importance and brain spatial information. For each regression model, we included *R* ^2^ and Cohen's *F* ^2^,^132^ the model's *F*‐value, model´s *p* value, and the statistical significance of the features. For space and clarity, following previous approaches,^127^, ^133^, ^134^ we reported the top three regression models for each set of outcomes, based on the highest *R* ^2^ values.

#### 2.6.2 XGBoost classifiers

We employed the XGBoost classifiers (see Supplementary Information S2) to evaluate the predictive value of country‐level inequality (measured by the Gini coefficient) alone and in combination with demographic (age, sex, and education) and cognitive (MMSE scores) factors on EEG metrics across the four outcome categories. Each set included several models, each corresponding to a specific ROI (see Table S6). The models were trained using an 80% training sample and then evaluated on a distinct 20% testing set, repeating the process for *k* = 10 iterations.^135^ Outcome variables were binarised using their median to ensure an equal class distribution. The feature importance, the ROC, the AUC, accuracy, precision, *F*1, and recall were calculated (see Supplementary Information S3). We reported the top 10 classification models for each set of outcomes, based on the highest AUC.

#### 2.6.3 Additional analyses

To explore the specificity of the Gini coefficient as a predictor of EEG metrics compared to other country‐level economic indicators, we conducted additional analyses including the gross domestic product (GDP) as a covariate in the classification models. These indicators were sourced from the updated country‐specific data provided on the World Bank's platform^136^ (https://databank.worldbank.org/). To address the combined effect of the Gini coefficient with demographic and cognitive factors, we conducted additional analyses including all variables simultaneously in the classification models. These models were run both with the full sample and with the subsample of participants who had cognitive measures. In addition, we conducted linear regressions to test the interactions between age and the Gini coefficient on the three EEG metrics mostly associated with structural inequality in each category, as identified in the regression models reported in Section 3.2.

### 2.7 Control for the effects of signal quality

The assessment of signal quality was conducted using the Overall Data Quality (OQD) index.^10^, ^127^, ^137^ The EEG signal was segmented into 1‐s epochs, each labelled as 1 (low quality) or 0 (high quality). The metric was discretised into four categories: ‘excellent’ for OQD ≥ 90%, ‘good’ for 90% > OQD ≥ 80%, ‘poor’ for 80% > OQD ≥ 60%, and ‘bad’ for OQD < 60%.^137^ We used four criteria to assess quality: (i) detection of epoch with constant amplitude and epochs containing missing or infinite voltages,^138^ (ii) detection of unusual high/low amplitude voltages across samples,^138^ (iii) high‐frequency noise,^139^ and (iv) low correlations between channels.^135^ OQD represents the percentage of EEG epochs with good quality, ranging from 0 (signals for which all epochs were classified as having poor quality) to 100 (signals for which all epochs were classified as having good quality).^137^ Logistic regressions with the Gini coefficient as outcome were carried out to determine if signal quality, the number of participants per centre, and the number of channels could predict the inequality values. In addition, to control for the ODQ, number of channels, sampling rate, and reference across sites, we conducted additional analyses including these parameters as covariates in all classification models. The ODQ was categorised as original suggested.^137^ The online reference was coded with non‐ordinal categories.

## 3 RESULTS

### 3.1 Structural income inequality is the most important predictor of EEG signals

We carried out hierarchical regressions to evaluate whether the Gini coefficient could predict EEG metrics, controlling for demographics and cognition, and to identify the EEG metrics most sensitive to the effects of inequality. We reported the three top hierarchical regression models aimed to predict EEG complexity, aperiodic spectral and power spectrum components, along with graph‐theoretic measures (Tables 2, 3, 4, 5). Inequality emerged as a significant and most robust predictor across all regressions, except for the connectivity models (*R* ^2^ values ≤ .05). Age and cognition were also significant predictors of the four metrics categories. Although less influential, sex significantly predicted complexity, power spectrum components, and network connectivity, whereas education only significantly predicted connectivity with very low *R* ^2^ values. In network connectivity metrics, inequality was significant only with the global efficiency of conditional mutual information.

**TABLE 2:** Top hierarchical regressions predicting EEG complexity components.

Wiener entropy (all ROIs)							
Features	Estimate	*t* value	*p* Value	*R* ^2^	*F* ^2^	*F*	*p* Value
Gini	−.3717	−11.551	<1e‐15	.13	.15	83.55	<1e‐15
Age	.1323	4.723	<1e‐15				
Gini	−.4169	−12.586	<1e‐15	.13	.15	80.26	<1e‐15
Education	−.046	−.49	.624				
Gini	−.381	−11.363	<1e‐15	.12	.13	70.32	<1e‐15
Sex	−.0345	−2.086	.037				
Gini	−.5194	−15.411	<1e‐15	.2996	.4278	125.98	<1e‐15
Cognition	.0242	.536	.592				

Permutation entropy (all ROIs)							
Features	Estimate	*t* value	*p* Value	*R* ^2^	*F* ^2^	*F*	*p* Value
Gini	−.4684	−10.419	<1e‐15	.12	.13	73.42	<1e‐15
Age	.2038	5.209	<1e‐15				
Gini	−.5274	−11.331	<1e‐15	.11	.12	64.39	<1e‐15
Education	−.1494	−1.132	.258				
Gini	−.519	−11.06	<1e‐15	.11	.12	64.22	<1e‐15
Sex	−.0279	−1.205	.228				
Gini	−.6629	−13.491	<1e‐15	.24	.32	94.82	<1e‐15
Cognition	−.0057	−.087	.931				

Fractional dimension (all ROIs)							
Features	Estimate	*t* value	*p* Value	*R* ^2^	*F* ^2^	*F*	*p* Value
Gini	−.2703	−9.499	<1e‐15	.08	.08	45.4	<1e‐15
Age	−.0401	−1.62	.105				
Gini	−.2841	−9.657	<1e‐15	.08	.09	46.78	<1e‐15
Education	−.0773	−.926	.354				
Gini	−.2412	−8.317	<1e‐15	.08	.09	47.54	<1e‐15
Sex	−.0588	−4.115	<1e‐15				
Gini	−.3295	−10.709	<1e‐15	.18	.22	65.43	<1e‐15
Cognition	.0707	1.72	.086				

**TABLE 3:** Top hierarchical regressions predicting EEG aperiodic spectral components.

Slope (right cingulate gyrus)							
Features	Estimate	*t* value	*p* Value	*R* ^2^	*F* ^2^	*F*	*p* Value
Gini	−1.2605	−9.689	<1e‐15	.11	.13	70.51	<1e‐15
Age	−.8747	−7.724	<1e‐15				
Gini	−1.1367	−8.781	<1e‐15	.08	.08	42.4	<1e‐15
Education	.5115	1.393	.164				
Gini	−1.0343	−7.885	<1e‐15	.06	.07	35.19	<1e‐15
Sex	−.1263	−1.953	.051				
Gini	−1.0858	−6.894	<1e‐15	.11	.13	37.55	<1e‐15
Cognition	.781	3.711	<1e‐15				

Slope (left parietal)							
Features	Estimate	*t* value	*p* Value	*R* ^2^	*F* ^2^	*F*	*p* Value
Gini	−1.1109	−8.919	<1e‐15	.12	.14	77.81	<1e‐15
Age	−1.0304	−9.502	<1e‐15				
Gini	−1.0591	−8.249	<1e‐15	.06	.07	35.28	<1e‐15
Education	.1118	.307	.759				
Gini	−.9129	−6.959	<1e‐15	.05	.05	28.69	7.35e‐13
Sex	−.1411	−2.182	.029				
Gini	−.9039	−6.034	<1e‐15	.09	.10	29.52	6.07e‐13
Cognition	.6818	3.406	.001				

Slope (right parietal)							
Features	Estimate	*t* value	*p* Value	*R* ^2^	*F* ^2^	*F*	*p* Value
Gini	−1.123	−8.842	<1e‐15	.12	.14	74.63	<1e‐15
Age	−1.0177	−9.205	<1e‐15				
Gini	−.9864	−7.47	<1e‐15	.05	.05	29.99	2.15e‐13
Education	.329	.878	.38				
Gini	−.8673	−6.49	<1e‐15	.04	.04	22.47	2.76e‐10
Sex	−.0616	−.934	.35				
Gini	−.8637	−5.672	<1e‐15	.09	.10	29.3	7.39e‐13
Cognition	.785	3.857	<1e‐15				

**TABLE 4:** Top hierarchical regressions predicting EEG power spectrum components.

Subj spec α_high_ relative power (left cingulate gyrus)							
Features	Estimate	*t* value	*p* Value	*R* ^2^	*F* ^2^	*F*	*p* Value
Gini	−.0005	−10.613	<1e‐15	.16	.19	101.99	<1e‐15
Age	−.0005	−10.488	<1e‐15				
Gini	−.0005	−8.601	<1e‐15	.07	.07	38.43	<1e‐15
Education	.0001	.36	.719				
Gini	−.0004	−7.896	<1e‐15	.07	.08	39.82	<1e‐15
Sex	−.0001	−3.238	.001				
Gini	−.0005	−7.411	<1e‐15	.13	.15	44.84	<1e‐15
Cognition	.0004	4.235	<1e‐15				

Subj spec α_high_ equivalent power (left cingulate gyrus)							
Features	Estimate	*t* value	*p* Value	*R* ^2^	*F* ^2^	*F*	*p* Value
Gini	−.0047	−10.613	<1e‐15	.16	.19	101.99	<1e‐15
Age	−.0041	−10.488	<1e‐15				
Gini	−.0044	−8.601	<1e‐15	.07	.07	38.43	<1e‐15
Education	.0005	.36	.719				
Gini	−.0039	−7.896	<1e‐15	.07	.08	39.82	<1e‐15
Sex	−.0008	−3.238	.001				
Gini	−.0042	−7.411	<1e‐15	.13	.15	44.84	<1e‐15
Cognition	.0032	4.235	<1e‐15				

Subj spec α_low_ relative power (left cingulate gyrus)							
Features	Estimate	*t* value	*p* Value	*R* ^2^	*F* ^2^	*F*	*p* Value
Gini	−.0005	−10.298	<1e‐15	.14	.16	88.11	<1e‐15
Age	−.0004	−9.285	<1e‐15				
Gini	−.0005	−8.41	<1e‐15	.07	.07	37.23	<1e‐15
Education	.0001	.624	.533				
Gini	−.0004	−7.761	<1e‐15	.07	.07	36.94	<1e‐15
Sex	−.0001	−2.791	.005				
Gini	−.0005	−7.679	<1e‐15	.14	.16	46.74	<1e‐15
Cognition	.0003	4.158	<1e‐15				

**TABLE 5:** Top hierarchical regressions predicting network organisation.

Global efficiency (CMI)							
Features	Estimate	*t* value	*p* Value	*R* ^2^	*F* ^2^	*F*	*p* Value
Gini	.034	3.176	.002	.15	.18	98.01	<1e‐15
Age	−.125	−13.339	<1e‐15				
Gini	.047	3.696	<1e‐15	.02	.02	10.49	3.1e‐05
Education	−.0802	−2.242	.025				
Gini	.0394	3.256	.001	.02	.02	10.08	4.62e‐05
Sex	−.0206	−3.499	<1e‐15				
Gini	.0596	4.004	<1e‐15	.03	.03	9.23	.000113
Cognition	.0466	2.343	.019				

### 3.2 Classification models

**FIGURE 2:**
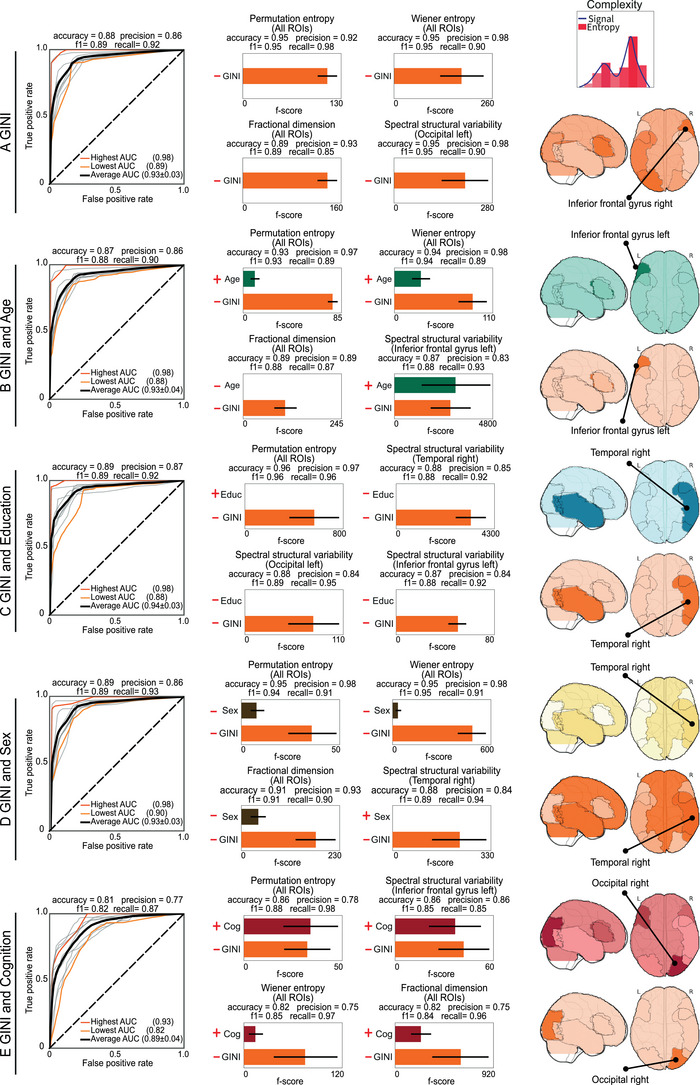
Inequality, demographics, and cognition as predictors of the EEG complexity. EEG complexity metrics were classified using five sets of classification models: (A) Gini, (B) Gini and age, (C) Gini and education, (D) Gini and sex, and (E) Gini and cognition. The symbols in the figure denote the orientation of the predictors in relation to the outcome. The **left panels** of each section show the ROC curves for the top 10 regression models (grey colour lines). The ROC curves with the lowest and highest area under curve (AUC) are denoted in light and dark orange, respectively, with the mean ROC highlighted by a black line. The average accuracy, precision, *F*1 score, and recall, are also provided. The **middle panels** display the feature importance for the top four classification models, alongside their performance metrics. The plus and minus signs on the left side of charts denote the direction of the correlation between the predictors and the EEG outcome. The **right panels** show brain topographical information from the top 10 classification models. The brain colours indicate the predictors being analysed and match the colour of the horizontal bars in middle panels). The darkness of the colours indicates the brain region's relevance for the classification, with darker tones representing greater relevance. The absence of bars in the central panels indicates that the predictors were not statistically significant in relation to the outcome.

**FIGURE 3:**
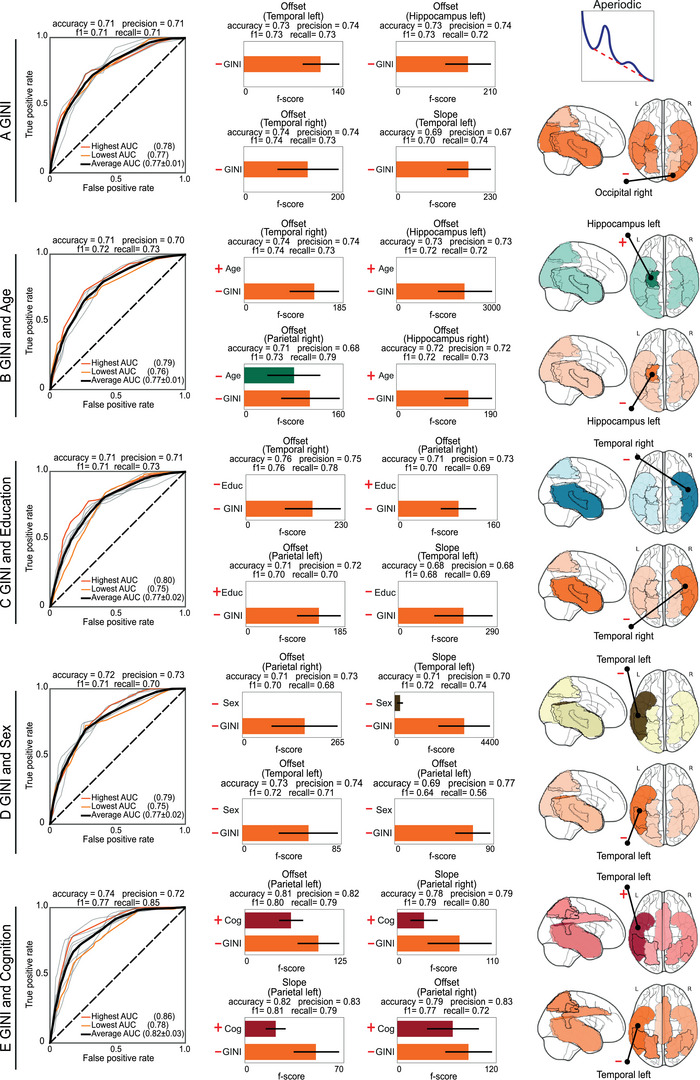
Inequality, demographics and cognition as predictors of aperiodic spectral components. The aperiodic spectral components were classified using the following predictors: (A) Gini, (B) Gini and age, (C) Gini and education, (D) Gini and sex and (E) Gini and cognition. The symbols in the figure denote the orientation of the predictors in relation to the outcome. The **left panels** of each section show the ROC curves for the top 10 regression models (grey colour lines). The ROC curves with the lowest and highest area under curve (AUC) are denoted in light and dark orange, respectively, with the mean ROC highlighted by a black line. The average accuracy, precision, *F*1 score, and recall are also provided. The **middle panels** display the feature importance for the top four classification models, alongside their performance metrics. The **right panels** show brain topographical information from the top 10 classification models. The brain colours correspond to the colours representing the predictors (horizontal bars in middle panels). Darker colours indicate a brain region's relevance in the models. The lack of bars in the central panels indicates that the predictors were not statistically significant with respect to the outcome.

**FIGURE 4:**
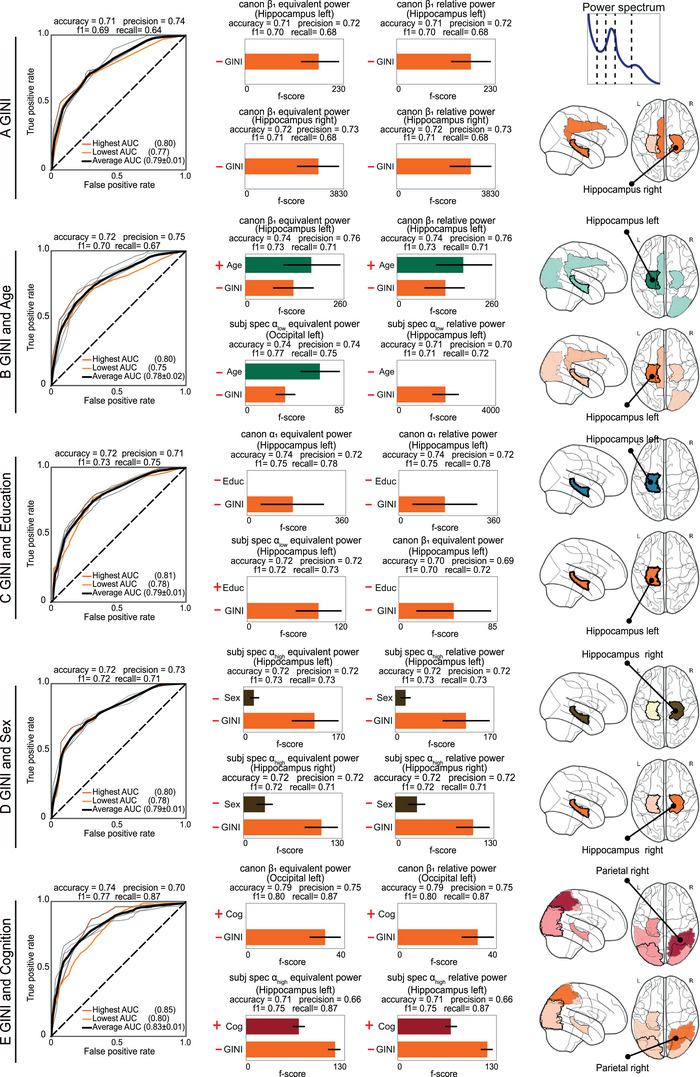
Inequality, demographics and cognition as predictors of power spectrum components. The power spectrum components were classified using the following predictors: (A) Gini, (B) Gini and age, (C) Gini and education, (D) Gini and sex, and (E) Gini and cognition. The symbols in the figure denote the orientation of the predictors in relation to the outcome. The left panels of each section show the ROC curves for the top 10 regression models (grey colour lines). The ROC curves with the lowest and highest area under curve (AUC) are denoted in light and dark orange, respectively, with the mean ROC highlighted by a black line. The average accuracy, precision, *F*1 score, and recall, are also provided. The middle panels display the feature importance for the top four classification models, alongside their performance metrics. The right panels show brain topographical information from the top 10 classification models. The brain colours correspond to the colours representing the predictors (horizontal bars in middle panels). Darker colours indicate a brain region's relevance in the models. The missing bars in the central panels indicate that the predictors were not statistically significant regarding the outcome.

**FIGURE 5:**
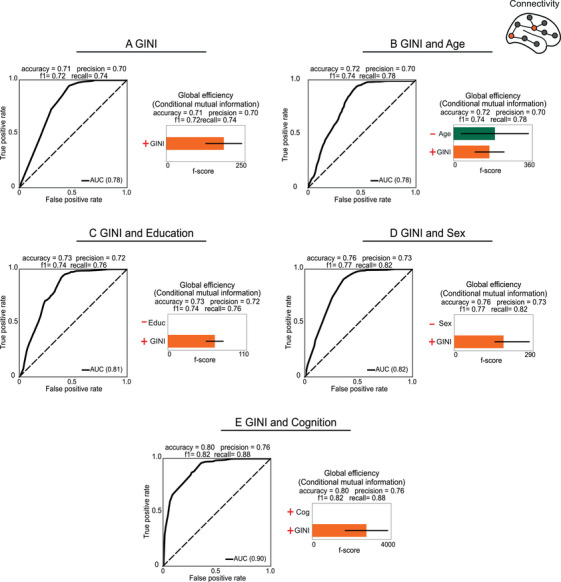
Inequality, demographics and cognition as predictors of graph‐theoretical measures. The graph‐theoretic measures were classified using the following predictors: (A) Gini, (B) Gini and age, (C) Gini and education, (D) Gini and sex, and (E) Gini and cognition. The symbols in the figure denote the orientation of the predictors in relation to the outcome. The **left panels** of each section show the ROC curves for the top 10 regression models (grey colour lines). The ROC curves are shown with the area under the curve (AUC) of the only outcome of graph‐theoretic measures that was significant in the results of Section 2.1. In each section, the **right panels** show the importance of the features. The absence of bars in the central panels indicates that the predictors were not statistically significant in relation to the outcome.

Utilising XGBoost classifiers, we assessed the Gini coefficient's ability to distinguish between high and low values of the EEG metrics identified with the hierarchical regressions. These classifiers included inequality as an independent factor and in combination with demographics and cognition, and reported feature importance and brain topographical information. The initial set of models focused on Gini as the sole predictor, with subsequent models incorporating Gini with an additional variable each time – age, education, sex or cognition – to examine their combined influence. The results for each set of classification models are displayed in Figures 2, 3, 4, 5. Each figure displays the mean ROC curve for the top 10 models with the highest AUC, alongside feature importance from the four most accurate models and relevant brain topographical information from the top 10 classification models.

#### 3.2.1 Structural income inequality is associated with poorer complexity measures

Higher inequality was consistently associated with decreased EEG complexity (Wiener entropy, permutation entropy, spectral structural variability and fractional dimension; Figure 2, middle panels). Inequality accurately classified complexity metrics individually [mean AUC of .93 (± .03)] and in combination with demographic or cognitive factors [mean AUC of .93 (± .03), with a maximum of .94 (± .03)] (Figure 2, left panels). (Figure 2B–E, middle panels). The four top models across all predictor sets revealed that inequality was the most influential predictor, surpassing demographics and cognition. Exceptions occurred for spectral structural variability in age models, and permutation entropy in cognition models (Figure 2B–E, middle panels). Education did not show statistical significance, and age and sex were not significant in the fractional dimension for all ROIs and spectral structural variability temporal right, respectively. The brain regions that most significantly reflected the associations between inequality and complexity metrics were in the inferior frontal gyrus and right occipital regions (calcarine fissure and surrounding cortex, cuneus, superior, middle, and inferior occipital gyri, lingual gyrus, Figures 2B–E, right panels).

#### 3.2.2 Structural income inequality is associated with lower aperiodic spectral activity

Higher levels of inequality were consistently associated with lower EEG aperiodic activity (offset and slope metrics; Figure 3, middle panels). Inequality accurately classified aperiodic spectral activity both individually [mean AUC of .77 (± .01)] and in combination with demographic or cognitive factors [mean AUC of .77 (± .02), with a maximum of .82 (± .03)] (Figure 3, left panels). Analysis of the top four models across all sets of predictors consistently revealed that inequality was a more influential predictor than demographics and cognition (Figure 3B–E, middle panels). Education did not demonstrate statistical significance, while age and sex were significant predictors only for the offset of the right parietal region and the slope of the left temporal region, respectively. The brain regions most significantly reflecting the associations between inequality and the aperiodic components were the left hippocampus, the bilateral temporal lobes (Heschl gyrus, superior, middle and inferior temporal gyri, middle and inferior temporal poles) and the right occipital lobe (calcarine fissure and surrounding cortex, cuneus, superior, middle, and inferior occipital gyri, lingual gyrus, Figure 3B–E, right panels).

#### 3.2.3 Structural income inequality is associated with lower spectral power

Higher inequality consistently predicted lower spectral power in the EEG beta and alpha band (Figure 4, left and middle panels). Inequality as the sole predictor [mean AUC of .79 (± .01)] or in combination with demographics and cognition achieved a high‐performance classification [mean AUC of .78 (± .02), maximum of .83 (± .01)] (Figure 4, left panels). The four best models in all predictor sets indicated that inequality was the most influential predictor, exceeding demographics and cognition, except for age models (Figure 4B–E, middle panels). Education did not exhibit statistical significance in any model. The models focusing on the hippocampus left and right, and parietal right (superior and inferior parietal gyri, angular gyrus, paracentral lobule) showed the highest accuracy in classifying power spectrum activity (Figure 4, right panels).

#### 3.2.4 Structural income inequality classifies network metrics

Inequality was significantly associated only with the global efficiency of conditional mutual information. Higher inequality was associated with increased network integration (global efficiency of conditional mutual information) (Figure 5A–E, right panels). Inequality accurately classified connectivity measures individually [AUC of .78] and when combined with demographics and cognition [AUC of .90]. Inequality emerged as a more influential predictor than demographics and cognition, except in the model incorporating age. Education, sex, and cognition did not show statistical significance (Figure 5B–E, right panels).

### 3.3 Effects of individual factors and other country‐level measures

To test the specificity of the Gini coefficient compared to other country‐level economic indicators, we conducted additional analyses including the GDP as a covariate in all classification models. The results were consistent with those reported above, confirming that structural income inequality was the most robust and systematic predictor of EEG metrics (Tables S6–S9).

To address the combined effect of the Gini coefficient with demographic and cognitive factors, we conducted additional analyses including all variables simultaneously in the classification models. These results confirmed that the Gini coefficient remains as the most robust predictor of EEG metrics, independent of individual demographic and cognitive factors (Tables S10–S13). We also tested the specific interactions between age and the Gini coefficient (Section 3.2). We found significant interactions for complexity metrics (WE and FD), aperiodic spectral components (slope), and global efficiency. These results are reported in Tables S14–S17.

### 3.4 Effects of signal quality and heterogeneity in EEG acquisition

Logistic regressions were conducted with the Gini coefficient as the outcome to assess whether signal quality, the number of participants per centre, or the number of channels could predict the inequality values. None of these factors accurately predicted inequality (Table S18). Additional analyses, including ODQ, number of channels, sampling rate, and online reference as covariates in classification models, confirmed that the results are not driven by these potential confounders (Tables S19–S22).

## 4 DISCUSSION

This study investigated the impact of structural income inequality on the brain's dynamics across a dataset of participants from countries with different levels of income inequality. Despite variability in samples, data collection methods, and EEG acquisition, structural inequality systematically predicted brain dynamics, proving to be a more crucial determinant of brain signals than individual‐level factors (demographics and cognition). Complexity and aperiodic activity metrics were the most sensitive in capturing the effects of structural inequality on brain function. Underscoring its biological embedding, structural income inequality was associated to changes in temporal dynamics linked with brain health conditions,^104^, ^140^, ^141^, ^142^, ^143^, ^144^, ^145^, ^146^, ^147^ potentially contributing to brain and cognitive aging.^52^, ^99^, ^100^, ^143^, ^148^, ^149^, ^150^ The current findings might challenge conventional neuroscience approaches that tend to overemphasise the influence of individual‐level factors (e.g., socioeconomic status, biological and psychological factors) on brain health outcomes, while neglecting structural factors in understanding brain function.^26^

Structural income inequality predicted multiple dimensions of brain signals. Complexity and aperiodicity metrics were most sensitive. Higher inequality was associated with decreased EEG complexity, characterised by more regular or repetitive patterns^99^, ^100^, ^148^ in frontal and occipital regions. Low EEG complexity is modulated by lower personal income,^23^ pathological conditions^104^ reduced information processing efficiency^93^, ^149^ and poorer cognitive performance.^52^, ^149^, ^150^ Structural income inequality predicted reduced aperiodic activity in the temporoposterior regions. This pattern of effects suggest an increase in random spontaneous (slope) and general spiking activity (offset),^151^ along with a decrease in signal‐to‐noise ratio.^152^ A flattened slope is typically observed in older adults,^99^, ^100^, ^148^ and in higher exposure to chronic stress.^153^ Decreases in slope ^96^, ^97^, ^98^ and offset^98^, ^154^, ^155^ correlate with worse cognitive performance. Structural inequality was also associated with reduced power (alpha and beta) in temporoposterior regions, a finding also reported in individuals from low socioeconomic status.^22^, ^24^ Alpha power also decreases with aging.^45^, ^99^ Higher structural inequality was also associated with increased integration of brain networks. Although this might be considered an unexpected result, increased global efficiency has been linked to hyperconnectivity in preclinical dementia.^156^ This modulation, together with decreased alpha power and a flattened slope, may suggest underlying neuronal hyperexcitability,^113^, ^157^ an early core feature of neurodegeneration.^156^, ^158^, ^159^ However, the associations between structural inequality and graph‐theoretical measures were the weakest and further research exploring specific brain networks is required. The temporoposterior regions, which are mainly associated with inequality, seem to be particularly linked to the cumulative burden throughout the lifespan. Gene expression^160^ and disruptions in adult neurogenesis^161^, ^162^, ^163^ makes these regions and the hippocampus especially vulnerable to neurodegeneration. Given that observed brain dynamics are linked to neurodegeneration,^104^, ^140^, ^141^, ^142^, ^143^, ^144^, ^145^, ^146^, ^147^ they could represent potential mechanisms through which structural inequality impacts brain aging.^52^, ^99^, ^100^, ^143^, ^148^, ^149^, ^150^ Overall, results provided convergent multimodal metrics of biologic embedding of structural income inequality^1^ primarily characterised by less complex signals, increased random asynchronous neural activity, and lower alpha and beta powers, particularly over temporoposterior hubs.

The influence of structural income inequality on brain signals surpassed that of a country's total economic output (GDP) and individual‐level factors, including demographics and cognition. Age and cognition showed relevance in some models, even surpassing the importance of inequality in some cases (complexity and power spectrum). Sex and education were the less influential predictors. The combined effects of the Gini coefficient and age suggest that higher inequality and older age increases random and spontaneous neural activity,^45^, ^164^ along with a reduction in alpha power.^45^, ^47^, ^99^ Significant interactions between the Gini coefficient and age suggest that the effects of structural income inequality on EEG complexity, aperiodic activity, and network integration may be exacerbated in older adults, potentially linking these effects to accelerated brain aging.^10^ In addition, age correlated with EEG complexity^47^, ^164^ and diminished network integration,^102^, ^165^, ^166^, ^167^ suggesting compromised network communication efficiency.^168^ Higher inequality and worse cognition, when combined, predicted less complexity in brain signals,^52^, ^149^, ^150^ an increase in random and spontaneous neural activity,^96^, ^97^, ^98^ and a decrease in alpha power.^143^ Additionally, better cognition predicted higher network integration.^51^, ^169^, ^170^ Consistent with the mixed findings regarding sex differences in EEG signals,^48^, ^49^, ^99^, ^159^, ^171^ this predictor had a minimal effect in the models, yet emerged as significant in some cases. Living in a highly unequal country and being female predicted less complex signals,^48^ reduced alpha power, and decreased network integration.^172^ Given that country‐level gender inequality is associated with brain structural differences,^37^ future studies should further explore the effects of macrostructural income and gender inequalities on EEG signals. Years of education showed a weak relationship with brain signals.^173^, ^174^, ^175^ While them have been used as an individual proxy for income inequality,^16^, ^43^, ^176^ our results suggest that the Gini coefficient is a stronger predictor of brain signals. Education could be used as a proxy for inequality in homogeneous populations; this relationship weakens among heterogeneous groups due to disparities.^177^, ^178^ Future studies should further test the contribution of individual‐level measures of socioeconomic inequality versus aggregate‐level measures, including more relevant measures of education quality.

Our results indicate that structural income inequality is associated with less efficient brain dynamics, transcending individual differences. In countries with lower inequality, economic resources enhance access to healthcare, leisure activities, social connections, and high‐quality education, all of which contribute positively to brain health.^1^, ^36^ Conversely, more unequal societies are exposed to cumulative environmental stressors, exposome and risk factors for brain diseases.^1^, ^11^, ^179^ This prolonged exposure can lead to an increased allostatic load, resulting in lasting alterations to brain structure,^1^, ^36^ physiological dysregulation, and a heightened risk of neurodegeneration.^180^ Other factors commonly associated with high inequality may further explain these outcomes, including poorer nutrition, lower levels of physical activity, less cognitively stimulating environments, and increased pollution.^1^, ^8^, ^181^ Given the significant influence of macro‐level factors on brain health, there is a pressing call for public policy interventions.^8^, ^38^, ^182^ Policies aimed at mitigating country‐level inequality and bolstering social support networks could markedly benefit the brain health of the most vulnerable groups. Moreover, our findings underscore the importance of inclusive research that addresses the wide‐ranging impacts of diversity and disparity on brain health.^1^, ^5^, ^8^ Additionally, results highlight the value of employing cost‐effective and scalable methods like EEG in researching inequality effects on brain signatures. EEG provides a practical tool for detecting physiological changes associated with early pathological conditions and dementia risk,^183^, ^184^ making it particularly useful for studying brain function in diverse and underrepresented populations.

Some limitations of our study should be acknowledged. First, although the Gini coefficient is a canonical metric of income structural inequality, it is not free of limitations.^185^, ^186^ Future research should incorporate additional macro‐level factors, such as air pollution, exposure to violence, or access to healthcare^11^, ^187^, ^188^ and the combined impact of structural inequality and key personal factors related to brain health, such as early‐life adversity, physical activity, and dietary habits.^11^, ^189^, ^190^ In addition, we assessed cognition using a screening tool. Although the MMSE has been widely utilised as a reliable measure of general cognitive state,^65^ it may not fully encompass the spectrum of cognitive abilities. We acknowledge the limitations of the MMSE as a tool for assessing cognition, particularly in healthy populations. However, the MMSE remains a valuable instrument for initial cognitive assessment in both clinical and research settings.^66^ We did not inquire about gender identities or education quality. Future studies should incorporate such measures to provide a comprehensive understanding of their associated brain signatures and interactions with structural inequality. Our study relies on EEG data. Future research should incorporate multi‐modal neuroimaging with higher spatial resolution to further explore the associations between country‐level structural income inequality and brain changes. Topographical interpretations from our analyses should be approached with caution due to EEG's spatial resolution limitation.

In conclusion, our findings show a relationship between structural income inequalities and brain temporal dynamics, highlighting the biological embedding of disparity in brain function. Considering that, individual and structural factors contribute to inequality and influence brain outcomes, results call for a reevaluation of traditional methodologies towards a more integrative approach that accounts for the interaction of both levels. Future research should delve into the underlying mechanisms, their moderating effects, and their impacts on different aspects of brain health. Our findings pave the way for neuroscience‐informed policies aimed at tackling structural inequalities in underserved and diverse populations.

## AUTHOR CONTRIBUTIONS

Sandra Baez and Hernan Hernandez: conception and design of the study; acquisition and analysis of data; drafting the manuscript and figures; Sebastian Moguilner, Jhosmary Cuadros, Hernando Santamaria‐Garcia, VicenteMedel, Joaquín Migeot, Josephine Cruzat, Pedro A. Valdes‐Sosa, Francisco Lopera, Alfredis González‐Hernández, Jasmin Bonilla‐Santos, Rodrigo A. Gonzalez‐Montealegre, Tuba Aktürk, Agustina Legaz, Sol Fittipaldi, Görsev G. Yener, Javier Escudero, Claudio Babiloni, Susanna Lopez, Robert Whelan, Alberto A Fernández Lucas, David Huepe, Marcio Soto‐Añari, Carlos Coronel‐Oliveros, Eduar Herrera, Daniel Abasolo, Ruaridh A. Clark, Bahar Güntekin, Claudia Duran‐Aniotz, Brian Lawlor, Enzo Tagliazucchi, Pavel Prado: conception and design of the study; acquisition of data, review and editing; Mario A. Parra: conception and design of the study; acquisition of data, review and editing, funding acquisition; Agustin Ibanez: conception and design of the study; acquisition of data, supervision, review and editing, funding acquisition.

## CONFLICT OF INTEREST STATEMENT

The authors declare that they have no known competing financial interests or personal relationships that could have appeared to influence the work reported in this paper.

## ETHICS STATEMENT

All procedures performed were in accordance with the ethical standards of the Helsinki Declaration and its later amendments. The Institutional Ethics Committee at each participating centre approved the study protocol, and all participants provided written informed consent.

## Supporting information



Supporting information

## Data Availability

The data and analysis codes are freely available at the following GitHub link https://github.com/euroladbrainlat/Structural‐inequality‐and‐brain‐dynamics‐across‐diverse‐samples. The data in the repository has been anonymised and preprocessed.
