# Supplementary material for: Structural inequality and temporal brain dynamics across diverse samples
Source: Clin Transl Med. 2024 Oct 3;14(10):e70032. doi: 10.1002/ctm2.70032 (PMC11447638; doi:10.1002/ctm2.70032)
Supplement: Supplementary file 1 — Supporting information [file CTM2-14-e70032-s001.docx]

**SUPPLEMENTARY MATERIALS**

**S1.** The study involved metrics such as mutual information, conditional mutual information, and organizational information (1-4). Mutual information (MI) quantifies the shared information between two random variables (or time series), revealing non-linear and non-monotonic relationships between these time series. It signifies how the uncertainty of one variable decreases when the information of the other is known. MI is a non-negative metric, registering a value of zero when the variables are statistically unrelated.

Conditional Mutual Information (CMI) quantifies the shared information between two random variables (or time series) while considering a third given variable. It represents the reduction in uncertainty of one variable when the information of another is known, taking into account the third variable. This third variable can be multivariate and is akin to the activity of all brain regions except the two for which mutual information is being calculated.

Organizational Information (O_info), an extension of Shannon's mutual information, provides a deeper understanding of the main attributes of multivariate systems, particularly those involving high-order interactions. O_info identifies situations dominated by redundancy, where identical information is shared among three or more variables. On the other hand, for systems leaning towards synergy, O_info notes that advanced high-order patterns cannot be deduced solely from low-order marginals.

**S2.** The XGBoost model leverages boosting techniques to create a potent ensemble of multiple decision trees. This model builds on an iterative approach where weak decision trees are sequentially improved, rectifying the errors from prior iterations to progressively boost accuracy (5, 6). XGBoost is distinguished by its efficient handling of missing data, capability to process large datasets, adaptability to various data distributions, and incorporation of regularization to avert overfitting. Additionally, it offers feature importance evaluations to pinpoint essential predictive factors. To optimize XGBoost's hyperparameters (7), Bayesian optimization was utilized, which involves setting a hyperparameter search domain, employing a surrogate probabilistic model, and iteratively suggesting new configurations, thereby effectively navigating the parameter space to find optimal settings.

**S3.** The ROC curve was utilized to visualize the trade-off between sensitivity and specificity in binary classification (8, 9). The AUC quantified the overall discriminative ability of the classification model. Accuracy measured the proportion of correctly classified instances, offering a comprehensive view of the model's performance. Precision focused on the accuracy of positive predictions, assessing the ratio of correctly predicted positive instances to the total predicted positives. The F1 Score, a harmonic mean of precision and recall, provided a balanced retrospective evaluation of the model's performance. Recall (sensitivity) gauged the model's past ability to correctly identify actual positive instances, emphasizing its sensitivity.

**Supplementary Table 1.**  Details on EEG recording features by center

| Center | Time | Eyes | Equipment | Channels | Ref. | Filter | Sampling rate |
| --- | --- | --- | --- | --- | --- | --- | --- |
| Centro de Neurociencias de Cuba (10) | 2-20 min | Closed | Digital Electroencephalogram system MEDICID | 64/128  Passive | Linked earlobes | 0.5-50 Hz | 200 Hz |
| BrainLat (11, 12) | 5-25 min | Closed | Biosemi ADBOX-MODELO MK2 | 128  Active | Mastoids | 0.16-100  Hz | 2048 Hz |
| Izmir University of Economics | 7-10 min | Closed 4 min  Open 4 min | BrainAmp | 32  Passive | A1 + A2  Electrodes | 0.03-70 Hz | 500 Hz |
| Trinity College Dublin | 3-12 min | Closed | Two Biosemi system | 264+7 sensors  Active | Average | 0.1-95 Hz | 512 Hz |
| Universidad de Antioquia | 5-8 min | Closed | Neuroscan Synamps 2 | 72  Passive | Vertex | 0.1-200 Hz | 200 Hz |
|  | 7-18 min | Closed | Neuroscan Synamps 2 | 72  Passive | Right mastoid | 0.1-200 Hz | 1024 Hz |
| Universidad de Sao Paulo (13) | 10-26 min | Closed | Braintech 3.0 | 21 | unknown | 1-60 Hz | 200 Hz |
| Sapienza Università di Roma | 8-14 min | Closed 5 min  Open 5 min | Brain Amp | 61  Passive | Fcz Electrode | Highpass 0.016 Hz | 1000 Hz |
| University of Strathclyde | 5-13 min | Closed | Two Biosemi system | 64  Active | Left and right mastoids | 0.1-35 Hz | 512 Hz |
| Istanbul Medipol University | 2-11 min | Closed 4 min  Open 4 min | Brain Amp | 30  Passive | A1 + A2 Electrodes | 0.01-250 Hz | 500 Hz |
| TAKEDA | 7-8 min | Closed | Two Biosemi system | 132  Active | Left and right mastoids |  | 1024 Hz |
| Open Neuro (14) | 5-21 min | Closed | Nihon Kohden EEG 2100 | 19 | A1 + A2 Electrodes | High filter 70 Hz | 500 Hz |

**Supplementary Table 2.** Complexity metrics

| **2.1. Fractal dimension (FD)** | **2.2. Permutation entropy (PE)** |
| --- | --- |
| $FD=a\left( NLD-NLD_{0} \right)^{k}$  where $NLD$ is the normalized length density, and $NLD_{0}$ is the initial value of *NLD* .The coefficients $a$, $k$, and $NLD_{0}$, were set to 1.9079, 0.18383 y 0.097178, respectively, as recommended in Kalauzi et al., 2009, (15). Additionally, $NLD$ was calculate as:  $NLD=\frac{1}{N}\sum_{i=2}^{N} \vert y_{n}\left( i \right)-y_{n}(i-1)\vert$  Where $y_{n}\left( i \right)$ represents the $ith$ signal sample after amplitude normalization. | Considering a time series $x_{t}$, where $t=1, \ldots, T$, and the embedded vector $X_{t}=[X_{t}+X_{t+l},\ldots,+X_{t+(n-1)}]$, where n is the embedding dimension and $l$ is the lag. After arranging the vector $X_{t}$ from smallest to largest, PE is defined as:  $PE=-\sum_{n=1}^{n!} p\left( \pi\right)\ln(p)$  where $f\left( \pi\right)$ represents the frequency of the time series and $p\left( \pi\right)=f\left( \pi\right)/(T-\left( n-1 \right)l)$ represents the relative frequency (16, 17). |
| **2.3. Wiener entropy (WE)** | **2.4. Spectral structure variability (SSV)** |
| $SFM(t)=log\frac{\left[ \prod_{i=1}^{N} S\left( t,f \right) \right]^{\frac{1}{N}}}{(\frac{1}{N})\sum_{i=1}^{N} S(t,f)}$  where N is the number of points in the Fourier transform, and $S\left( t,f \right)$ is the associated power at each frequency component. Finally, the WE is calculated as the average of$SFM(t)$. | $SFM(t)=log\frac{\left[ \prod_{i=1}^{N} S\left( t,f \right) \right]^{\frac{1}{N}}}{(\frac{1}{N})\sum_{i=1}^{N} S(t,f)}$  where N is the number of points in the Fourier transform, and $S\left( t,f \right)$ is the associated power at each frequency component. Finally, the SSV is calculated as the variance of $SFM(t)$. |

**Supplementary Table 3.** Spectral and aperiodic metrics, and entropy EEG outcomes used for the linear regression models

| Type of parameter | Metric |  | ROI |
| --- | --- | --- | --- |
| Spectral | Relative power density | $\delta$ | Orbital gyrus, inferior frontal gyrus, middle frontal gyrus, superior frontal gyrus, hippocampal regions and amygdala, insula |
|  | Equivalent percent power |  |  |
|  | Relative power density | $\theta$ |  |
|  | Equivalent percent power |  |  |
|  | Relative power density | $\alpha$ | Hippocampal regions and amygdala, parietal lobe, occipital lobe |
|  | Equivalent percent power |  |  |
|  | Relative power density | $\beta$ | Hippocampal regions and amygdala, insula, cingulate cortex, parietal lobe, temporal lobe, occipital lobe |
|  | Equivalent percent power |  |  |
|  | Relative power density | $\gamma$ | Orbital gyrus, inferior frontal gyrus, hippocampal regions and amygdala, insula, temporal lobe, |
|  | Equivalent percent power |  |  |
|  | Individual α frequency peak | | Orbital gyrus, inferior frontal gyrus, middle frontal gyrus, superior frontal gyrus, hippocampal regions and amygdala, insula, , occipital lobe |
|  | $\theta$ - α transition frequency | | Orbital gyrus, inferior frontal gyrus, middle frontal gyrus, superior frontal gyrus, hippocampal regions and amygdala, insula |
| Aperiodic | Slope | of the 1/f fit | Hippocampal regions and amygdala, cingulate cortex, parietal lobe, temporal lobe, occipital lobe |
|  | Knee |  |  |
|  | Offset |  |  |

**Supplementary Table 4.** Connectivity metrics

| **4.1. Connection weight** | **4.2. Weighted shortest path length** |
| --- | --- |
| The strength of the link between nodes is denoted by the connection weight, where $w_{ij}$ specifically refers to the connection weight between node *i* and node *j*. | $d_{ij}= \sum_{a_{uv}\in gi\leftrightarrow j} f(w_{uv})$  where *f* maps weight to length, and $gi\leftrightarrow j$ denotes the shortest weighted path between *i* and *j*. |
| **4.3. Weighted characteristic path length** | **4.4. Number of triangles** |
| $L= \frac{1}{n}\sum_{i} \frac{\sum_{j\neq i} d_{ij}}{n-1}$  where *n* denotes the number of nodes in the network, and $d_{ij}$ is the length of the shortest path between node *i* and node *j*. | $t_{i}= \frac{1}{2}{\sum_{j,h} \left( w_{ij}w_{ih}w_{jh} \right)}^{\frac{1}{3}}$  where $t_{i}$ is the weighted geometric mean of triangles around *i, j* and *h* are indices indicating the neighboring nodes of *i* in the weighted matrix. |
| **4.3. Weighted degree** | **4.6. Weighted clustering coefficient** |
| $k_{i}= \sum_{j} w_{ij}$  where $k_{i}$ is the degree of a node *i*. | $C= \frac{1}{n}\sum_{i} \frac{2t_{i}}{k_{i}(k_{i}-1)}$  where $t_{i}$ is the number of triangles around *i* and $k_{i}$ is the weighted degree of a node *i*. |
| **4.7. Weighted global efficiency** | **4.9. Weighted transitivity** |
| $E= \frac{1}{n(n-1)}\sum_{i\neq j} \frac{1}{d_{ij}}$  where *n* denotes the number of nodes in the network, and $d_{ij}$ is the length of the shortest path between node *i* and node *j*. | $T= \frac{\sum_{i} 2t_{i}}{\sum_{i} k_{i}(k_{i}-1)}$  where $t_{i}$ is the number of triangles around *i* and $k_{i}$ is the weighted degree of a node *i*. |
| **4.10. Weighted density** | **4.11. Small-worldness (**$\boldsymbol{\sigma}$**)** |
| $D = \frac{\sum_{i\neq j} w_{ij}}{n(n-1)}$  where $w_{ij}$ denotes the weight of the connection between node *i* and node *j*, and n is the total number of nodes in the network. | $\sigma= \frac{C/C_{r}}{L/L_{r}}$  where $C$ is the clustering coefficient in the network, $C_{r}$ is the expected clustering coefficient in a random graph with the same number of nodes and edges, $L$ is the observed characteristic path length of the network, and ($L_{r}$) is the expected characteristic path length in the random graph. |

**Supplementary Table 5.** Regions of interest selected for the estimation of EEG parameters

| Region of interest (ROI) | Regions comprising the ROI |
| --- | --- |
| Orbital Gyrus | Superior, middle, and inferior orbital gyri |
| Inferior Frontal Gyrus | Opercular and triangular parts of the inferior frontal gyrus |
| Middle Frontal Gyrus | Middle frontal gyrus |
| Superior Frontal Gyrus | Precentral gyrus, superior, and medial superior frontal gyri, gyrus rectus, supplementary motor area |
| Hippocampal Regions and Amygdala | Hippocampus, para-hippocampal area, amygdala |
| Insula | Rolandic operculum, insula |
| Cingulate Cortex | Anterior, middle, and posterior cingulate |
| Parietal Lobe | Superior and inferior parietal gyri, angular gyrus, paracentral lobule |
| Temporal Lobe | Heschl gyrys, superior, middle, and inferior temporal gyri, middle and inferior temporal poles |
| Occipital Lobe | Calcarine fissure and surrounding cortex, cuneus, superior, middle, and inferior occipital gyri, lingual gyrus |

**Supplementary Table 6.** Top classification models predicting complexity components including GDP as covariate.

| Top hierarchical regressions predicting EEG complexity components | | | | | | | | | | | |
| --- | --- | --- | --- | --- | --- | --- | --- | --- | --- | --- | --- |
| **Permutation Entropy**  AUC = 1.0, Accuracy = 0.95, Precision = 0.94, F1 = 0.95, Recall = 0.96 | | | | |  | **Permutation Entropy**  AUC = 0.91, Accuracy = 0.8, Precision = 0.72, F1 = 0.83, Recall = 0.97 | | | | | |
| **Gini** | **Age** | **Education** | **Sex** | **GDP** |  | **Gini** | **Age** | **Education** | **Sex** | **Cog** | **GDP** |
| 27.6 (5.95) | 12.4 (4.4) | 2.80 (1.87) | 1.0 (<0.01) | 39.0 (7.6) |  | 37.0 (18.54) | 16.22 (13.93) | 8.20 (2.74) | 1.0 (<0.01) | 8.25 (6.73) | 42.1 (14.97) |
|  | | | | |  |  | | | | | |
| **Wiener Entropy**  AUC = 0.98, Accuracy = 0.97, Precision = 0.99, F1 = 0.97, Recall = 0.94 | | | | |  | **Wiener Entropy**  AUC = 0.92, Accuracy = 0.8, Precision = 0.72, F1 = 0.83, Recall = 0.98 | | | | | |
| **Gini** | **Age** | **Education** | **Sex** | **GDP** |  | **Gini** | **Age** | **Education** | **Sex** | **Cog** | **GDP** |
| 21.4 (5.62) | 9.20 (4.78) | 1.57 (0.53) | 2.60 (1.14) | 37.50 (10.93) |  | 23.9 (21.09) | 16.3 (24.82) | 10.2 (10.46) | 2.0 (<0.01) | 11.0 (9.58) | 30.3 (19.22) |
|  | | | | |  |  | | | | | |
| **Fractional Dimension**  AUC = 0.95, Accuracy = 0.93, Precision = 0.93, F1 = 0.93, Recall = 0.93 | | | | |  | **Fractional Dimension**  AUC = 0.9, Accuracy = 0.79, Precision = 0.72, F1 = 0.82, Recall = 0.97 | | | | | |
| **Gini** | **Age** | **Education** | **Sex** | **GDP** |  | **Gini** | **Age** | **Education** | **Sex** | **Cog** | **GDP** |
| 18.60 (8.46) | 19.80 (7.45) | 4.33  (2.0) | 2.56 (1.94) | 30.40 (11.27) |  | 23.0 (17.25) | 11.33 (6.76) | 11.3 (6.63) | 1.5 (0.71) | 7.7 (4.11) | 29.3 (12.78) |
|  | | | | |  |  | | | | | |
| **SSV Inferior Frontal Gyrus Right**  AUC = 0.91, Accuracy = 0.86, Precision = 0.81, F1 = 0.87, Recall = 0.94 | | | | |  | **SSV Inferior Frontal Gyrus Right**  AUC = 0.88, Accuracy = 0.81, Precision = 0.76, F1 = 0.82, Recall = 0.9 | | | | | |
| **Gini** | **Age** | **Education** | **Sex** | **GDP** |  | **Gini** | **Age** | **Education** | **Sex** | **Cog** | **GDP** |
| 19.90 (6.74) | 10.40 (5.52) | 5.40 (3.63) | 4.0 (<0.01) | 39.50 (10.44) |  | 19.5 (9.7) | 10.2 (11.56) | 4.20 (1.93) | 2.75 (2.55) | 4.33 (1.15) | 26.5 (8.49) |

Cog = cognition, Gini = Gini index, GDP = Gross domestic product

**Supplementary Table 7.** Top classification models predicting aperiodic spectral components including GDP as covariate.

| Top hierarchical regressions predicting EEG aperiodic spectral components | | | | | | | | | | | |
| --- | --- | --- | --- | --- | --- | --- | --- | --- | --- | --- | --- |
| **Offset Hippocampus Left**  AUC = 0.83, Accuracy = 0.78, Precision = 0.77, F1 = 0.79, Recall = 0.82 | | | | |  | **Offset Hippocampus Left**  AUC = 0.83, Accuracy = 0.73, Precision = 0.67, F1 = 0.77, Recall = 0.91 | | | | | |
| **Gini** | **Age** | **Education** | **Sex** | **GDP** |  | **Gini** | **Age** | **Education** | **Sex** | **Cog** | **GDP** |
| 32.4 (10.66) | 27.8 (12.09) | 9.0  (7.62) | 2.60 (2.61) | 48.8 (16.01) |  | 27.8 (13.1) | 24.6 (16.09 | 19.0 (13.03) | 2.4 (1.67) | 6.5 (5.42) | 24.1 (8.02) |
|  | | | | |  |  | | | | | |
| **Offset Temporal Left**  AUC = 0.81, Accuracy = 0.76, Precision = 0.75, F1 = 0.77, Recall = 0.79 | | | | |  | **Offset Temporal Left**  AUC = 0.84, Accuracy = 0.73, Precision = 0.68, F1 = 0.76, Recall = 0.87 | | | | | |
| **Gini** | **Age** | **Education** | **Sex** | **GDP** |  | **Gini** | **Age** | **Education** | **Sex** | **Cog** | **GDP** |
| 38.2 (11.15) | 26.40 (11.31) | 23.40 (9.0) | 3.50 (1.87) | 59.0 (16.9) |  | 26.7 (6.85) | 18.1 (5.13) | 13.4 (6.28) | 3.8 (2.04) | 8.3 (3.77) | 26.1 (4.58) |
|  | | | | |  |  | | | | | |
| **Offset Temporal Right**  AUC = 0.8, Accuracy = 0.76, Precision = 0.74, F1 = 0.77, Recall = 0.8 | | | | |  | **Offset Temporal Right**  AUC = 0.81, Accuracy = 0.71, Precision = 0.67, F1 = 0.74, Recall = 0.84 | | | | | |
| **Gini** | **Age** | **Education** | **Sex** | **GDP** |  | **Gini** | **Age** | **Education** | **Sex** | **Cog** | **GDP** |
| 30.2 (11.8) | 43.9 (30.93) | 17.7 (18.64) | 5.70 (4.9) | 50.0 (13.75) |  | 28.4 (6.8) | 22.0 (4.0) | 14.6 (8.04) | 4.2 (1.93) | 5.8 (3.39) | 22.9 (4.12) |
|  | | | | |  |  | | | | | |
| **Slope Temporal left**  AUC = 0.8, Accuracy = 0.73, Precision = 0.71, F1 = 0.74, Recall = 0.77 | | | | |  | **Slope Temporal left**  AUC = 0.82, Accuracy = 0.73, Precision = 0.75, F1 = 0.72, Recall = 0.7 | | | | | |
| **Gini** | **Age** | **Education** | **Sex** | **GDP** |  | **Gini** | **Age** | **Education** | **Sex** | **Cog** | **GDP** |
| 30.2 (7.21) | 28.1 (11.53) | 14.3 (9.86) | 4.0 (<0.01) | 43.8 (11.36) |  | 21.0 (5.85) | 25.10 (6.98) | 8.80 (5.43) | 3.14 (1.35) | 7.90 (2.42) | 32.7 (7.35) |

Cog = cognition, Gini = Gini index, GDP = Gross domestic product

**Supplementary Table 8.** Top classification models predicting power spectrum components including GDP as covariate.

| Top hierarchical regressions predicting EEG power spectrum components | | | | | | | | | | | |
| --- | --- | --- | --- | --- | --- | --- | --- | --- | --- | --- | --- |
| **canon β_1_ equivalent power**  **Hippocampus Left**  AUC = 0.8, Accuracy = 0.73, Precision = 0.75, F1 Score = 0.72, Recall = 0.7 | | | | |  | **canon β_1_ equivalent power**  **Hippocampus Left**  AUC = 0.82, Accuracy = 0.71, Precision = 0.7, F1 Score = 0.73, Recall = 0.77 | | | | | |
| **Gini** | **Age** | **Education** | **Sex** | **GDP** |  | **Gini** | **Age** | **Education** | **Sex** | **Cog** | **GDP** |
| 24.80 (7.41) | 30.90 (11.81) | 10.00 (5.52) | 2.00 (1.73) | 36.90 (9.52) |  | 47.50 (15.83) | 39.10 (16.12) | 27.60 (12.74) | 12.30 (5.4) | 14.90 (6.21) | 17.70 (8.37) |
|  | | | | |  |  | | | | | |
| **canon β_1_ relative power**  **Hippocampus Left**  AUC = 0.8, Accuracy = 0.73, Precision = 0.75, F1 Score = 0.72, Recall = 0.7 | | | | |  | **canon β_1_ relative power**  **Hippocampus Left**  AUC = 0.82, Accuracy = 0.71, Precision = 0.7, F1 Score = 0.73, Recall = 0.77 | | | | | |
| **Gini** | **Age** | **Education** | **Sex** | **GDP** |  | **Gini** | **Age** | **Education** | **Sex** | **Cog** | **GDP** |
| 24.80 (7.41) | 30.90 (11.81) | 10.00 (5.52) | 2.00 (1.73) | 36.90 (9.52) |  | 47.50 (15.83) | 39.10 (16.12) | 27.60 (12.74) | 12.30 (5.4) | 14.90 (6.21) | 17.70 (8.37) |
|  | | | | |  |  | | | | | |
| **canon β_1_ equivalent power**  **Hippocampus Right**  AUC = 0.82, Accuracy = 0.74, Precision = 0.75, F1 Score = 0.73, Recall = 0.72 | | | | |  | **canon β_1_ equivalent power**  **Hippocampus Right**  AUC = 0.77, Accuracy = 0.72, Precision = 0.66, F1 Score = 0.76, Recall = 0.89 | | | | | |
| **Gini** | **Age** | **Education** | **Sex** | **GDP** |  | **Gini** | **Age** | **Education** | **Sex** | **Cog** | **GDP** |
| 33.40 (15.44) | 31.70 (16.87) | 22.40 (19.39) | 7.20 (6.07) | 44.40 (15.82) |  | 42.20 (13.77) | 27.10 (17.29) | 22.30 (8.45) | 4.40 (1.9) | 12.10 (5.28) | 11.80 (10.83) |
|  | | | | |  |  | | | | | |
| **canon β_1_ relative power**  **Hippocampus Right**  AUC = 0.82, Accuracy = 0.74, Precision = 0.75, F1 Score = 0.73, Recall = 0.72 | | | | |  | **canon β_1_ relative power**  **Hippocampus Right**  AUC = 0.77, Accuracy = 0.72, Precision = 0.66, F1 Score = 0.76, Recall = 0.89 | | | | | |
| **Gini** | **Age** | **Education** | **Sex** | **GDP** |  | **Gini** | **Age** | **Education** | **Sex** | **Cog** | **GDP** |
| 33.40 (15.44) | 31.70 (16.87) | 22.40 (19.39) | 7.20 (6.07) | 44.40 (15.82) |  | 42.20 (13.77) | 27.10 (17.29) | 22.30 (8.45) | 4.40 (1.9) | 12.10 (5.28) | 11.80 (10.83 |

Cog = cognition, Gini = Gini index, GDP = Gross domestic product

**Supplementary Table 9.** Top classification models predicting all ROIs network organization including GDP as covariate.

| Top hierarchical regressions predicting all ROIs network organization | | | | | | | | | | | |
| --- | --- | --- | --- | --- | --- | --- | --- | --- | --- | --- | --- |
| **Global Efficiency (CMI)**  AUC = 0.89, Accuracy = 0.79, Precision = 0.74, F1 Score = 0.81, Recall = 0.89 | | | | |  | **Global Efficiency (CMI)**  AUC = 0.82, Accuracy = 0.74, Precision = 0.76, F1 Score = 0.73, Recall = 0.7 | | | | | |
| **Gini** | **Age** | **Education** | **Sex** | **GDP** |  | **Gini** | **Age** | **Education** | **Sex** | **Cog** | **GDP** |
| 29.20 (7.36) | 23.20 (8.11) | 6.50  (2.8) | 1.50 (1.0) | 30.8 (6.48) |  | 33.70 (11.08) | 25.90 (17.81) | 12.90 (10.6) | 2.75 (2.05) | 3.88 (2.47) | 26.6 (7.79) |

Cog = cognition, Gini = Gini index, GDP = Gross domestic product

**Supplementary Table 10.** Top classification models predicting complexity components including all variables.

| **Permutation Entropy**  AUC = 0.99, Accuracy = 0.97, Precision = 1.0, F1 = 0.97, Recall = 0.95 | | | |  | **Permutation Entropy**  AUC = 0.92, Accuracy = 0.8, Precision = 0.73,  F1 = 0.83, Recall = 0.97 | | | | |
| --- | --- | --- | --- | --- | --- | --- | --- | --- | --- |
| **Gini** | **Age** | **Education** | **Sex** |  | **Gini** | **Age** | **Education** | **Sex** | **Cognition** |
| 78.9 (37.04) | 16.3 (19.03) | 7.20 (8.46) | 2.80  (3.19) |  | 59.9  (36.98) | 31.10 (21.89) | 10.50 (7.0) | 3.20 (1.14) | 9.20 (5.25) |
| **Wiener Entropy**  AUC = 0.98, Accuracy = 0.97, Precision = 0.99, F1 = 0.97, Recall = 0.96 | | | |  | **Wiener Entropy**  AUC = 0.94, Accuracy = 0.8, Precision = 0.71,  F1 = 0.83, Recall = 0.99 | | | | |
| **Gini** | **Age** | **Education** | **Sex** |  | **Gini** | **Age** | **Education** | **Sex** | **Cognition** |
| 80.5 (27.13) | 12.8 (9.31) | 6.60  (4.77) | 2.70  (2.06) |  | 57.1 (27.53) | 26.6 (13.1) | 11.1 (4.93) | 1.17 (0.41) | 8.4 (3.75) |
| **Fractional Dimension**  AUC = 0.95, Accuracy = 0.94, Precision = 0.94, F1 = 0.94, Recall = 0.95 | | | |  | **Fractional Dimension**  AUC = 0.93, Accuracy = 0.81, Precision = 0.77, F1 Score = 0.82, Recall = 0.9 | | | | |
| **Gini** | **Age** | **Education** | **Sex** |  | **Gini** | **Age** | **Education** | **Sex** | **Cognition** |
| 79.4 (24.33) | 25.10 (10.27) | 11.10 (6.56) | 2.20  (1.55) |  | \| 54.1 (27.81) \| \| --- \| | 19.8 (13.94) | 11.1 (5.34) | 2.88 (2.75) | 14.4 (5.52) |
| **Fractional Dimension Right**  AUC = 0.89, Accuracy = 0.85, Precision = 0.79, F1 Score = 0.86, Recall = 0.94 | | | |  | **SSV Inferior Frontal Gyrus Right**  AUC = 0.83, Accuracy = 0.75, Precision = 0.71,  F1 = 0.77, Recall = 0.83 | | | | |
| **Gini** | **Age** | **Education** | **Sex** |  | **Gini** | **Age** | **Education** | **Sex** | **Cognition** |
| 117.4 (81.9) | 52.70 (79.27) | 31.5 (36.74) | 4.57  (6.05) |  | 59.70 (31.66) | 56.70 (43.68) | 21.90 (16.91) | 6.0 (6.06) | 9.70 (9.35) |

**Supplementary Table 11.** Top classification models predicting aperiodic spectral components including all variables.

| **Offset Hippocampus Left**  AUC = 0.82, Accuracy = 0.79, Precision = 0.77, F1 Score = 0.8, Recall = 0.84 | | | |  | **Offset Hippocampus Left**  AUC = 0.8, Accuracy = 0.71, Precision = 0.65, F1 = 0.75, Recall = 0.91 | | | | |
| --- | --- | --- | --- | --- | --- | --- | --- | --- | --- |
| **Gini** | **Age** | **Education** | **Sex** |  | **Gini** | **Age** | **Education** | **Sex** | **Cognition** |
| 86.4 (22.33) | 31.4 (20.26) | 18.1 (10.48) | 3.88  (4.29) |  | 59.70 (35.67) | 40.0 (39.99) | 42.9 (33.63) | 7.10 (7.03) | 14.0 (13.93) |
| **Offset Temporal Left**  AUC = 0.82, Accuracy = 0.77, Precision = 0.74, F1 Score = 0.79, Recall = 0.84 | | | |  | **Offset Temporal Left**  AUC = 0.84, Accuracy = 0.73, Precision = 0.69, F1 = 0.75, Recall = 0.83 | | | | |
| **Gini** | **Age** | **Education** | **Sex** |  | **Gini** | **Age** | **Education** | **Sex** | **Cognition** |
| 91.9 (21.59) | 30.4 (15.78) | 19.1 (10.24) | 3.60  (2.07) |  | 47.0  (20.24) | 29.6 (15.85) | 20.7 (9.92) | 4.38 (2.67) | 9.0 (7.12) |
| **Offset Temporal Right**  AUC = 0.81, Accuracy = 0.76, Precision = 0.74, F1 Score = 0.77, Recall = 0.81 | | | |  | **Offset Temporal Right**  AUC = 0.82, Accuracy = 0.72, Precision = 0.67, F1 = 0.75, Recall = 0.85 | | | | |
| **Gini** | **Age** | **Education** | **Sex** |  | **Gini** | **Age** | **Education** | **Sex** | **Cognition** |
| 82.4 (18.03) | 41.3 (19.41) | 16.1 (12.1) | 1.83  (0.41) |  | 46.80 (20.01) | 45.50 (24.79) | 25.10 (14.93) | 4.80 (4.44) | 20.20 (9.22) |
| **Slope Temporal Left**  AUC = 0.82, Accuracy = 0.72, Precision = 0.7, F1 Score = 0.74, Recall = 0.79 | | | |  | **Slope Temporal Left**  AUC = 0.84, Accuracy = 0.73, Precision = 0.69, F1 = 0.75, Recall = 0.82 | | | | |
| **Gini** | **Age** | **Education** | **Sex** |  | **Gini** | **Age** | **Education** | **Sex** | **Cognition** |
| 107.7 (27.53) | 48.4 (21.84) | 28.7 (16.52) | 6.50  (2.84) |  | 38.90  (6.19) | 29.80 (8.13) | 16.60 (7.12) | 4.60 (2.01) | 9.40 (1.43) |

**Supplementary Table 12.** Top classification models predicting power spectrum components including all variables.

| **canon β_1_ equivalent power**  **Hippocampus Left**  AUC = 0.82, Accuracy = 0.73, Precision = 0.73, F1 = 0.72, Recall = 0.72 | | | |  | **canon β_1_ equivalent power**  **Hippocampus Left**  AUC = 0.83, Accuracy = 0.71, Precision = 0.68, F1 = 0.73, Recall = 0.78 | | | | |
| --- | --- | --- | --- | --- | --- | --- | --- | --- | --- |
| **Gini** | **Age** | **Education** | **Sex** |  | **Gini** | **Age** | **Education** | **Sex** | **Cognition** |
| 86.20 (24.08) | 42.80 (27.79) | 20.20 (20.68) | 3.17  (2.71) |  | 54.40 (22.96) | 34.7 (12.56) | 23.10 (9.56) | 11.20 (4.73) | 16.20 (12.29) |
| **canon β_1_ relative power**  **Hippocampus Left**  AUC = 0.82, Accuracy = 0.73, Precision = 0.73, F1 = 0.72, Recall = 0.72 | | | |  | **canon β_1_ relative power**  **Hippocampus Left**  AUC = 0.83, Accuracy = 0.71, Precision = 0.68, F1 = 0.73, Recall = 0.78 | | | | |
| **Gini** | **Age** | **Education** | **Sex** |  | **Gini** | **Age** | **Education** | **Sex** | **Cognition** |
| 86.20 (24.08) | 42.80 (27.79) | 20.20 (20.68) | 3.17  (2.71) |  | 54.40 (22.96) | 34.7 (12.56) | 23.1 (9.56) | 11.2 (4.73) | 16.2 (12.29) |
| **canon β_1_ equivalent power**  **Hippocampus Right**  AUC = 0.79, Accuracy = 0.73, Precision = 0.73, F1 Score = 0.73, Recall = 0.74 | | | |  | **canon β_1_ equivalent power**  **Hippocampus Right**  AUC = 0.78, Accuracy = 0.71, Precision = 0.66, F1 = 0.74, Recall = 0.86 | | | | |
| **Gini** | **Age** | **Education** | **Sex** |  | **Gini** | **Age** | **Education** | **Sex** | **Cognition** |
| 81.20 (32.26) | 34.80 (21.54) | 17.60 (21.48) | 5.11  (6.25) |  | 59.30 (25.32) | 39.60 (29.81) | 29.40 (17.06) | 4.40 (3.03) | 26.8 (12.54) |
| **canon β_1_ relative power**  **Hippocampus Right**  AUC = 0.79, Accuracy = 0.73, Precision = 0.73, F1 Score = 0.73, Recall = 0.74 | | | |  | **canon β_1_ relative power**  **Hippocampus Right**  AUC = 0.78, Accuracy = 0.71, Precision = 0.66, F1 = 0.74, Recall = 0.86 | | | | |
| **Gini** | **Age** | **Education** | **Sex** |  | **Gini** | **Age** | **Education** | **Sex** | **Cognition** |
| 81.20 (32.26) | 34.80 (21.54) | 17.60 (21.48) | 5.11  (6.25) |  | 59.30 (25.32) | 39.60 (29.81) | 29.40 (17.06) | 4.40 (3.03) | 26.8 (12.54) |

**Supplementary Table 13.** Top classification models predicting all ROIs network organization including all variables.

| **Global Efficiency (CMI)**  AUC = 0.89, Accuracy = 0.83, Precision = 0.76, F1 = 0.85, Recall = 0.98 | | | |  | **Global Efficiency (CMI)**  AUC = 0.8, Accuracy = 0.73, Precision = 0.72, F1 = 0.73, Recall = 0.73 | | | | |
| --- | --- | --- | --- | --- | --- | --- | --- | --- | --- |
| **Gini** | **Age** | **Education** | **Sex** |  | **Gini** | **Age** | **Education** | **Sex** | **Cognition** |
| 66.80 (16.41) | 20.90 (11.55) | 14.00 (8.5) | 2.0 (<0.001) |  | 72.50  (37.25) | 43.40 (32.91) | 20.20 (9.46) | 2.50 (1.91) | 11.60 (9.5) |

**Supplementary Table 14.** Results of linear regressions assessing the effects of age, the Gini coefficient, and their interaction on the top three complexity metrics.

| Wiener entropy (R² = 0.14, F = 71.96, p<1e^-15^) | | | |
| --- | --- | --- | --- |
|  | **Estimate** | **t** | **P value** |
| Age | -0.071 | -0.92 | 0.36 |
| Gini | -0.578 | -6.96 | 5.22e^-12^ |
| Age:Gini | 0.385 | 2.69 | 0.007 |
| Permutation entropy (R² = 0.12, F = 61.43, p<1e^-15^) | | | |
|  | **Estimate** | **t** | **P value** |
| Age | 0.075 | 0.70 | 0.49 |
| Gini | -0.586 | -5.07 | 4.56e^-07^ |
| Age:Gini | 0.218 | 1.09 | 0.28 |
| Fractional dimension (R² = 0.08, F = 40.43, p<1e^-15^) | | | |
|  | **Estimate** | **t** | **P value** |
| Age | -0.257 | -3.85 | 0.0001 |
| Gini | -0.482 | -6.73 | 2.47e^-11^ |
| Age:Gini | 0.417 | 3.38 | 0.0007 |

**Supplementary Table 15.** Results of linear regressions assessing the effects of age, the Gini coefficient, and their interaction on the top three aperiodic spectral components.

| Slope (Right cingulate gyrus) [R² = 0.11, F = 58.27, p<1e^-15^] | | | |
| --- | --- | --- | --- |
|  | **Estimate** | **t** | **P value** |
| Age | 0.100 | 0.33 | 0.74 |
| Gini | -0.140 | -0.43 | 0.67 |
| Age:Gini | -1.866 | -3.33 | 0.001 |
| Slope (Left parietal) [R² = 0.12, F = 61.11, p<1e^-15^] | | | |
|  | **Estimate** | **t** | **P value** |
| Age | -0.389 | -1.32 | 0.19 |
| Gini | -0.399 | -1.27 | 0.20 |
| Age:Gini | -1.175 | -2.15 | 0.03 |
| Slope (Right parietal) [R² = 0.11, F = 58.10, p<1e^-15^] | | | |
|  | **Estimate** | **t** | **P value** |
| Age | -0.092 | -0.31 | 0.76 |
| Gini | -0.065 | -0.20 | 0.84 |
| Age:Gini | -1.728 | -3.11 | 0.002 |

**Supplementary Table 16.** Results of linear regressions assessing the effects of age, the Gini coefficient, and their interaction on the top three power spectrum components.

| Subj spec αhigh relative power (Left cingulate) [R² = 0.14, F = 73.12, p<1e^-15^] | | | |
| --- | --- | --- | --- |
|  | **Estimate** | **t** | **P value** |
| Age | -0.00486 | -4.075 | 4.86e^-05^ |
| Gini | -0.00515 | -4.03 | 6.02e^-05^ |
| Age:Gini | 0.00096 | 0.44 | 0.66 |
| Subj spec αlow relative power (Left cingulate) [R² = 0.13, F = 66.47 p<1e^-15^] | | | |
|  | **Estimate** | **t** | **P value** |
| Age | -0.00037 | -2.95 | 0.003 |
| Gini | -0.00042 | -3.16 | 0.002 |
| Age:Gini | -0.0001 | -0.45 | 0.65 |

**Supplementary Table 17.** Results of linear regressions assessing the effects of age, the Gini coefficient, and their interaction on the top brain network organization metric

| Global efficiency (CMI) [R² = 0.16, F = 85.98, p<1e^-15^] | | | |
| --- | --- | --- | --- |
|  | **Estimate** | **t** | **P value** |
| **Age** | -0.26 | -9.62 | 3.03e^-21^ |
| **Gini** | -0.11 | -3.69 | 0.0002 |
| **Age:Gini** | 0.26 | 5.21 | 2.16e^-07^ |

**Supplementary Table 18.** Results for logistic regression using ODQ, number of participants, and number of channels to predict Gini

| **AUC = 0.55 (±0.03), p = 0.19, pseudo-R² = 0.003 (±0.001)** | |
| --- | --- |
| Feature | Estimate |
| ODQ | 0.73734 |
| **AUC = 0.52 (±0.02), p = 0.65, pseudo-R² = 0.0007 (±0.0001)** | |
| Feature | Estimate |
| Number of participants | 0.20947 |
| **AUC = 0.63 (±0.05), p = <1e-15, pseudo-R² = 0.06 (±0.003)** | |
| Feature | Estimate |
| Number of channels | 2.56 |

**Supplementary Table 19.** Top classification models predicting EEG complexity using signal quality (ODQ), number of channels, online reference, and sampling frequency as covariates.

|  | **Gini** | **Sampling** |  | **Gini** | **Sampling** |
| --- | --- | --- | --- | --- | --- |
| **Permutation Entropy**  AUC = 0.99, Accuracy = 0.94, Precision = 0.93, F1 = 0.94, Recall = 0.96 | 48.5 (5.1) | 0 (-) | **Wiener Entropy**  AUC = 0.97, Accuracy = 0.94, Precision = 0.99, F1 = 0.94, Recall = 0.9 | 47.3 (15.11) | 0 (-) |
| **Fractional Dimension**  AUC = 0.90, Accuracy = 0.86, Precision = 0.89, F1 = 0.86, Recall = 0.83 | 39.1 (3.25) | 20.3 (13.98) | **SSV Inferior Frontal Gyrus**  AUC = 0.87, Accuracy = 0.81, Precision = 0.78, F1 = 0.82, Recall = 0.87 | 53.6 (13.89) | 0 (-) |
|  | **Gini** | **Reference** |  | **Gini** | **Reference** |
| **Permutation Entropy**  AUC = 0.99, Accuracy = 0.94, Precision = 0.93, F1 = 0.94, Recall = 0.95 | 41.9 (5.55) | 30.1 (4.31) | **Wiener Entropy**  AUC = 0.97, Accuracy = 0.94, Precision = 0.99, F1 = 0.93, Recall = 0.88 | 38.2 (4.26) | 0 (-) |
| **Fractional Dimension**  AUC = 0.9, Accuracy = 0.86, = 0.88, F1 Score = 0.86, Recall = 0.84 | 42.8 (4.05) | 0 (-) | **SSV Inferior Frontal Gyrus**  AUC = 0.88, Accuracy = 0.82, Precision = 0.78, F1 = 0.83, Recall = 0.89 | 50.7 (3.74) | 0 (-) |
|  | **Gini** | **Channels** |  | **Gini** | **Channels** |
| **Permutation Entropy**  AUC = 0.99, Accuracy = 0.95, Precision = 0.92, F1 = 0.95, Recall = 0.97 | 46.4 (5.17) | 42.3 (12.28) | **Wiener Entropy**  AUC = 0.96, Accuracy = 0.94, Precision = 0.98, F1 = 0.93, Recall = 0.89 | 37.8 (3.36) | 18.0 (15.16) |
| **Fractional Dimension**  AUC = 0.89, Accuracy = 0.86, Precision = 0.88, F1 = 0.86, Recall = 0.84 | 41.8 (4.29) | 5.90 (2.81) | **Fractional Dimension**  AUC = 0.88, Accuracy = 0.86, Precision = 0.87, F1 = 0.86, Recall = 0.84 | 43.0 (4.29) | 21.3 (13.4) |
|  | **Gini** | **ODQ** |  | **Gini** | **ODQ** |
| **Permutation Entropy**  AUC = 0.98, Accuracy = 0.94, Precision = 0.96, F1 = 0.94, Recall = 0.93 | 48.0 (4.85) | 26.9 (8.36) | **Wiener Entropy**  AUC = 0.99, Accuracy = 0.98, Precision = 1.0, F1 = 0.98, Recall = 0.95 | 40.3 (5.62) | 18.6 (4.81) |
| **Fractional Dimension**  AUC = 0.98, Accuracy = 0.91, Precision = 0.89, F1 = 0.92, Recall = 0.94 | 51.5 (3.81) | 21.6 (7.11) | **SSV Inferior Frontal Gyrus**  AUC = 0.89, Accuracy = 0.82, Precision = 0.78, F1 = 0.84, Recall = 0.9 | 60.6 (19.63) | 16.3 (7.42) |

**Supplementary Table 20.** Top classification models predicting EEG aperiodic spectral components, using signal quality (ODQ), number of channels, online reference, and sampling frequency as covariates.

|  | **Gini** | **Sampling** |  | **Gini** | **Sampling** |
| --- | --- | --- | --- | --- | --- |
| **Offset Hippocampus Left**  AUC = 0.79, Accuracy = 0.76, Precision = 0.77, F1 = 0.76, Recall = 0.74 | 37.9 (4.58) | 7.60 (2.72) | **Offset Temporal Left**  AUC = 0.75, Accuracy = 0.72, Precision = 0.74, F1 = 0.71, Recall = 0.68 | 43.0 (2.79) | 6.90 (2.73) |
| **Offset Temporal Right**  AUC = 0.75, Accuracy = 0.72, Precision = 0.74, F1 = 0.7, Recall = 0.67 | 52.5 (17.95) | 10.1 (6.51) | **Slope Temporal Left**  AUC = 0.73, Accuracy = 0.67, Precision = 0.67, F1 = 0.67, Recall = 0.67 | 59.3 (45.86) | 11.7 (8.19)) |
|  | **Gini** | **Reference** |  | **Gini** | **Reference** |
| **Offset Hippocampus Left**  AUC = 0.8, Accuracy = 0.76, Precision = 0.75, F1 = 0.76, Recall = 0.76 | 50.1 (4.89) | 33.7 (10.98) | **Offset Temporal Left**  AUC = 0.77, Accuracy = 0.73, Precision = 0.74, F1 = 0.72, Recall = 0.71 | 51.0 (6.48) | 31.6 (12.22) |
| **Offset Temporal Right**  AUC = 0.74, Accuracy = 0.72, Precision = 0.73, F1 = 0.72, Recall = 0.7 | 50.6 (5.82) | 29.2 (13.51)) | **Slope Temporal Left**  AUC = 0.74, Accuracy = 0.68, Precision = 0.67, F1 = 0.69, Recall = 0.72 | 52.6 (5.72) | 0 (-) |
|  | **Gini** | **Channels** |  | **Gini** | **Channels** |
| **Offset Hippocampus Left**  AUC = 0.79, Accuracy = 0.75, Precision = 0.73, F1 Score = 0.76, Recall = 0.79 | 51.3 (20.02) | 22.9 (9.09) | **Offset Temporal Left**  AUC = 0.77, Accuracy = 0.74, Precision = 0.73, F1 Score = 0.74, Recall = 0.75 | 48.6 (5.58) | 20.3 (7.59) |
| **Offset Temporal Right**  AUC = 0.73, Accuracy = 0.72, Precision = 0.74, F1 Score = 0.7, Recall = 0.67 | 45.7 (5.96) | 7.60 (2.95) | **Slope Temporal Left**  AUC = 0.73, Accuracy = 0.68, Precision = 0.67, F1 Score = 0.68, Recall = 0.7 | 57.5 (26.4) | 23.5 (10.32) |
|  | **Gini** | **ODQ** |  | **Gini** | **ODQ** |
| **Offset Hippocampus Left**  AUC = 0.85, Accuracy = 0.78, Precision = 0.74, F1 Score = 0.79, Recall = 0.85 | 46.4 (13.16) | 24.0 (8.42) | **Offset Temporal Left**  AUC = 0.85, Accuracy = 0.79, Precision = 0.75, F1 Score = 0.8, Recall = 0.87 | 53.8 (4.61) | 25.2 (6.97) |
| **Offset Temporal Right**  AUC = 0.84, Accuracy = 0.77, Precision = 0.74, F1 Score = 0.79, Recall = 0.85 | 51.7 (3.97) | 20.5 (2.12) | **Slope Temporal Left**  AUC = 0.84, Accuracy = 0.78, Precision = 0.75, F1 Score = 0.8, Recall = 0.85 | 85.2 (57.87) | 36.9 (21.71) |

**Supplementary Table 21.** Top classification models predicting EEG power spectrum components, using signal quality (ODQ), number of channels, online reference, and sampling frequency as covariates.

|  | **Gini** | **Sampling** |  | **Gini** | **Sampling** |
| --- | --- | --- | --- | --- | --- |
| **canon β_1_ equivalent power Hippocampus Left**  AUC = 0.76, Accuracy = 0.67, Precision = 0.66, F1 = 0.67, Recall = 0.68 | 43.3 (5.5) | 0 (-) | **canon β_1_ relative power**  **Hippocampus Left**  AUC = 0.76, Accuracy = 0.67, Precision = 0.66, F1 Score = 0.67, Recall = 0.68 | 43.3 (5.5) | 0 (-) |
| **canon β_1_ equivalent power Hippocampus Right**  AUC = 0.76, Accuracy = 0.68, Precision = 0.7, F1 = 0.66, Recall = 0.63 | 59.3 (25.36) | 0 (-) | **canon β_1_ equivalent power Hippocampus Right**  AUC = 0.76, Accuracy = 0.68, Precision = 0.7, F1 = 0.66, Recall = 0.63 | 59.3 (25.36) | 0 (-) |
|  | **Gini** | **Reference** |  | **Gini** | **Reference** |
| **canon β_1_ equivalent power Hippocampus Left**  AUC = 0.76, Accuracy = 0.67, Precision = 0.66, F1 Score = 0.67, Recall = 0.68 | 44.1 (5.8) | 36.6 (14.9) | **canon β_1_ relative power**  **Hippocampus Left**  AUC = 0.76, Accuracy = 0.67, Precision = 0.66, F1 Score = 0.67, Recall = 0.68 | 44.1 (5.8) | 36.6 (14.9) |
| **canon β_1_ equivalent power Hippocampus Right**  AUC = 0.76, Accuracy = 0.69, Precision = 0.72, F1 Score = 0.66, Recall = 0.62 | 67.9 (33.92) | 49.8 (16.55) | **canon β_1_ equivalent power Hippocampus Right**  AUC = 0.76, Accuracy = 0.69, Precision = 0.72, F1 Score = 0.66, Recall = 0.62 | 67.9 (33.92) | 49.8 (16.55) |
|  | **Gini** | **Channels** |  | **Gini** | **Channels** |
| **canon β_1_ equivalent power Hippocampus Left**  AUC = 0.75, Accuracy = 0.67, Precision = 0.66, F1 Score = 0.67, Recall = 0.68 | 40.4 (3.34) | 21.9 (11.86) | **canon β_1_ relative power**  **Hippocampus Left**  AUC = 0.75, Accuracy = 0.67, Precision = 0.66, F1 Score = 0.67, Recall = 0.68 | 40.4 (3.34) | 21.9 (11.86) |
| **canon β_1_ equivalent power Hippocampus Right**  AUC = 0.75, Accuracy = 0.67, Precision = 0.66, F1 Score = 0.67, Recall = 0.68 | 54.2 (9.0) | 0 (-) | **canon β_1_ equivalent power Hippocampus Right**  AUC = 0.75, Accuracy = 0.67, Precision = 0.66, F1 Score = 0.67, Recall = 0.68 | 54.2 (9.0) | 0 (-) |
|  | **Gini** | **ODQ** |  | **Gini** | **ODQ** |
| **canon β_1_ equivalent power Hippocampus Left**  AUC = 0.85, Accuracy = 0.79, Precision = 0.75, F1 Score = 0.8, Recall = 0.86 | 39.1 (2.56) | 13.20 (2.94) | **canon β_1_ relative power**  **Hippocampus Left**  AUC = 0.85, Accuracy = 0.79, Precision = 0.75, F1 Score = 0.8, Recall = 0.86 | 39.1 (2.56) | 13.20 (2.94) |
| **canon β_1_ equivalent power Hippocampus Right**  AUC = 0.82, Accuracy = 0.74, Precision = 0.72, F1 Score = 0.75, Recall = 0.79 | 59.0 (21.18) | 19.5 (9.19) | **canon β_1_ equivalent power Hippocampus Right**  AUC = 0.82, Accuracy = 0.74, Precision = 0.72, F1 Score = 0.75, Recall = 0.79 | 59.0 (21.18) | 19.5 (9.19) |

**Supplementary Table 22.** Top classification models predicting all ROIs network organization, using signal quality (ODQ), number of channels, online reference, and sampling frequency as covariates.

| Top hierarchical regressions predicting all ROIs network organization | | | | | |
| --- | --- | --- | --- | --- | --- |
|  | **Gini** | **Sampling** |  | **Gini** | **Reference** |
| **Global Efficiency (CMI)**  AUC = 0.81, Accuracy = 0.76, Precision = 0.7, F1 = 0.8, Recall = 0.94 | 35.8 (2.39) | 18.0 (7.23) | **Global Efficiency (CMI)**  AUC = 0.79, Accuracy = 0.72, Precision = 0.67, F1 = 0.76, Recall = 0.88 | 36.2 (0.63) | 36.9 (10.93) |
|  | **Gini** | **Channels** |  | **Gini** | **ODQ** |
| **Global Efficiency (CMI)**  AUC = 0.82, Accuracy = 0.75, Precision = 0.72, F1 = 0.76, Recall = 0.8 | 42.5 (8.29) | 45.4 (14.61) | **Global Efficiency (CMI)**  AUC = 0.75, Accuracy = 0.7, Precision = 0.64, F1 Score = 0.74, Recall = 0.88 | 35.7 (16.36) | 0 (-) |

**Supplementary references**

1. Herzog R, Rosas FE, Whelan R, Fittipaldi S, Santamaria-Garcia H, Cruzat J, et al. Genuine high-order interactions in brain networks and neurodegeneration. Neurobiol Dis. 2022;175:105918.

2. Prado P, Moguilner S, Mejia JA, Sainz-Ballesteros A, Otero M, Birba A, et al. Source space connectomics of neurodegeneration: One-metric approach does not fit all. Neurobiol Dis. 2023;179:106047.

3. Ince RA, Giordano BL, Kayser C, Rousselet GA, Gross J, Schyns PG. A statistical framework for neuroimaging data analysis based on mutual information estimated via a gaussian copula. Hum Brain Mapp. 2017;38(3):1541-73.

4. Cover TM, Thomas JA. Entropy, relative entropy and mutual information. Elements of Information Theory. 2nd edition ed2005. p. 13-55.

5. Kaufmann T, van der Meer D, Doan NT, Schwarz E, Lund MJ, Agartz I, et al. Common brain disorders are associated with heritable patterns of apparent aging of the brain. Nature Neuroscience. 2019;22(10):1617-23.

6. Chen T, Guestrin C. XGBoost: A Scalable Tree Boosting System. Proceedings of the 22nd ACM SIGKDD International Conference on Knowledge Discovery and Data Mining; San Francisco, California, USA: Association for Computing Machinery; 2016. p. 785–94.

7. Feurer M, Hutter F. Hyperparameter Optimization. In: Hutter F, Kotthoff L, Vanschoren J, editors. Automated Machine Learning: Methods, Systems, Challenges. Cham: Springer International Publishing; 2019. p. 3-33.

8. Zhe Hui H, Jane C, Dawn T. What is an ROC curve? Emergency Medicine Journal. 2017;34(6):357.

9. Hajian-Tilaki K. Receiver Operating Characteristic (ROC) Curve Analysis for Medical Diagnostic Test Evaluation. Caspian J Intern Med. 2013;4(2):627-35.

10. Bosch-Bayard J, Galan L, Aubert Vazquez E, Virues Alba T, Valdes-Sosa PA. Resting State Healthy EEG: The First Wave of the Cuban Normative Database. Front Neurosci. 2020;14:555119.

11. Legaz A, Prado P, Moguilner S, Baez S, Santamaria-Garcia H, Birba A, et al. Social and non-social working memory in neurodegeneration. Neurobiol Dis. 2023;183:106171.

12. Birba A, Fittipaldi S, Cediel Escobar JC, Gonzalez Campo C, Legaz A, Galiani A, et al. Multimodal neurocognitive markers of naturalistic discourse typify diverse neurodegenerative diseases. Cerebral Cortex. 2021;32(16):3377-91.

13. Huggins CJ, Escudero J, Parra MA, Scally B, Anghinah R, Vitória Lacerda De Araújo A, et al. Deep learning of resting-state electroencephalogram signals for three-class classification of Alzheimer's disease, mild cognitive impairment and healthy ageing. J Neural Eng. 2021;18(4).

14. Miltiadous A, Tzimourta KD, Afrantou T, Ioannidis P, Grigoriadis N, Tsalikakis DG, et al. A Dataset of Scalp EEG Recordings of Alzheimer&rsquo;s Disease, Frontotemporal Dementia and Healthy Subjects from Routine EEG. Data. 2023;8(6):95.

15. Kalauzi A, Bojić T, Rakić L. Extracting complexity waveforms from one-dimensional signals. Nonlinear Biomedical Physics. 2009;3(1):8.

16. Ouyang G, Li J, Liu X, Li X. Dynamic characteristics of absence EEG recordings with multiscale permutation entropy analysis. Epilepsy Research. 2013;104(3):246-52.

17. Li X, Ouyang G, Richards DA. Predictability analysis of absence seizures with permutation entropy. Epilepsy Res. 2007;77(1):70-4.
